# Coronary heart disease policy models: a systematic review

**DOI:** 10.1186/1471-2458-6-213

**Published:** 2006-08-18

**Authors:** Belgin Unal, Simon Capewell, Julia Alison Critchley

**Affiliations:** 1 Department of Public Health, Dokuz Eylul University School of Medicine, Izmir, Turkey; 2 Department of Public Health, University of Liverpool, UK; 3 Institute of Health and Society, Newcastle University, UK

## Abstract

**Background:**

The prevention and treatment of coronary heart disease (CHD) is complex. A variety of models have therefore been developed to try and explain past trends and predict future possibilities. The aim of this systematic review was to evaluate the strengths and limitations of existing CHD policy models.

**Methods:**

A search strategy was developed, piloted and run in MEDLINE and EMBASE electronic databases, supplemented by manually searching reference lists of relevant articles and reviews. Two reviewers independently checked the papers for inclusion and appraisal. All CHD modelling studies were *included *which addressed a defined population and reported on one or more key outcomes (deaths prevented, life years gained, mortality, incidence, prevalence, disability or cost of treatment).

**Results:**

In total, 75 articles describing 42 models were included; 12 (29%) of the 42 models were micro-simulation, 8 (19%) cell-based, and 8 (19%) life table analyses, while 14 (33%) used other modelling methods. Outcomes most commonly reported were cost-effectiveness (36%), numbers of deaths prevented (33%), life-years gained (23%) or CHD incidence (23%). Among the 42 models, 29 (69%) included one or more risk factors for primary prevention, while 8 (19%) just considered CHD treatments. Only 5 (12%) were comprehensive, considering both risk factors and treatments. The six best-developed models are summarised in this paper, all are considered in detail in the appendices.

**Conclusion:**

Existing CHD policy models vary widely in their depth, breadth, quality, utility and versatility. Few models have been calibrated against observed data, replicated in different settings or adequately validated. Before being accepted as a policy aid, any CHD model should provide an explicit statement of its aims, assumptions, outputs, strengths and limitations.

## Background

Improving population health through effective interventions remains a fundamental challenge for policy makers. Decision-makers at the population and individual levels each need to choose the 'best intervention' for a specific health problem. However, limitations on resources, time and information can make this decision process difficult. This is particularly true for cardiovascular disease, its diversity of manifestations and wealth of effective interventions are potentially complex and confusing. Assessing the potential population benefit of a health intervention requires consideration of many elements including disease prevalence and population characteristics, effectiveness and cost [1]. Clinical trials will never provide all the answers, particularly since their study groups are restricted with inclusion and exclusion criteria; therefore generalisation is always an issue [2]. Weinstein has usefully defined a model as, "a logical mathematical framework that permits the integration of facts and values to produce outcomes of interest to clinicians and decision makers" or, alternatively as: "an analytical methodology that accounts for events over time and across populations based on data drawn from primary or secondary sources"[3].

Models have been increasingly used in policy making and resource allocation, because they permit policy makers to simulate the effects of different scenarios within a population[4] and hence examine future policy options.

An appropriate model for coronary heart disease (CHD) could thus potentially allow users to simultaneously consider all the key issues when evaluating diverse options for intervention.

A remarkably wide variety of CHD policy models exist. Some simply consider risk factors alone[5], while others include selected cardiovascular treatments[1,6,7] secondary prevention such as cholesterol lowering treatment[8] or estimates of general practice workload [9]. However, model construction and development is complex and difficult. Thus, few CHD models have attempted a comprehensive consideration of all standard treatments and all major risk factors[10]. Furthermore, the variable quality and utility of different models may not always be appreciated.

In this paper we have systematically reviewed and evaluated the strengths and limitations of existing CHD policy models. Critiquing and providing a comprehensive overview of all CHD models is a challenging task. Although it is useful to have a broad summary of the similarities and differences of models, it is virtually impossible to capture the complexity and subtlety of a range of models in a single paper. However, this paper represents our best efforts, and will hopefully serve as an overview and introduction to this complex field. It will also provide sources and routes to further information on specific models.

## Methods

For this systematic review, we defined a CHD policy model as any mathematical tool that may help to explain or predict the outcome of CHD interventions: specific treatments or cardiovascular risk factor changes or the implementation of a new strategy at a population level.

### Search strategy

A search strategy was developed, piloted and run in MEDLINE and EMBASE electronic databases on 12^th ^May 2003, supplemented by screening reference lists of relevant articles and reviews. Electronic searching within the databases included 'coronary heart disease or synonyms' and 'model or synonyms' as key words. Both key words and MeSH headings were used (Appendix 1) [see Additional file 1]. The search strategy was validated using ten key papers already known to the authors; all ten papers were captured by the search strategy. The search initially identified 4,531 articles and checking the references identified a further 17. All the records were imported to 'Reference Manager'. By checking the titles and abstracts for the terms 'model', 'coronary heart disease' or 'population', the number of articles was reduced to 275. Two reviewers (BU, SC) independently checked the titles and abstracts of all papers initially identified, and then independently screened the articles for inclusion and appraisal. Agreement between the two reviewers was good (Kappa = 0.76).

### Inclusion and exclusion criteria

Any CHD modelling study was included if it reported on a key outcome (deaths prevented, life years gained, prevention cost, treatment cost, mortality, prevalence, incidence or disability) in a defined population (community, region or country). Models were excluded if they simply described animals, cell lines, clinical series, cohorts or estimates of individual risk. Decision analytic models were also not included in this review since they mostly focused on clinical decision making in individuals, rather than population benefits.

**Figure 1:**
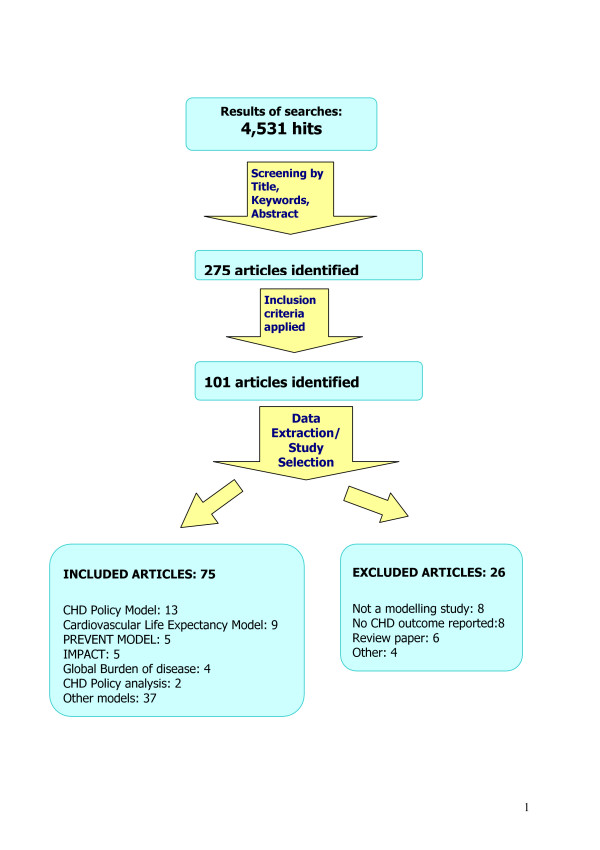
Flowchart of search strategy for CHD policy models.

Figure 1 illustrates the flowchart for the search and review process. Excluded articles are listed in Appendix 4 [see Additional file 1]. In total, 75 articles were included for critical appraisal and 200 articles were excluded.

### Data extraction and assessment of model quality

A pre-piloted form was used for data extraction (Appendix 2) [see Additional file 1]. Articles were categorised according to the specific models that they described. Each paper was then critically appraised by two reviewers using explicit quality criteria. There are no universally accepted lists of appropriate quality criteria for model papers. However reviews by Weinstein [11], and Edwards [12], and recent guidelines from the International Society for Pharmacoeconomics and Outcomes Research (ISPOR) [3] have suggested useful quality criteria. Using these sources [3,11,12], we created, piloted and refined a grading system, based on sensitivity, validity and transparency of the model (Appendix 3) [see Additional file 1].

### Quality measures

Papers were evaluated on the basis of whether a sensitivity analysis was carried out, the validity was checked, data quality was reported, illustrative examples were provided, assumptions stated, if model was potentially available to the reader (transparency), and if potential limitations such as assumptions, confounding, lag times and competing causes were specified or discussed. The model evaluation was based on authors' reporting on that specific item in the related articles. A model was considered as comprehensive if it included multiple coronary heart disease categories, and a range of treatments and major risk factors.

### Limitations of the review

Our search was limited to English language articles or abstracts. Publication bias can be an issue since our search was mostly based on electronic search of biomedical databases. The grey literature was not formally searched; however, we contacted key people who were working in this field.

## Results

From 4,531 initial papers, a total of 75 articles describing 42 different CHD policy models were finally included (Figure 1). Each was originally used to address one or more specific health policy questions and all were based on large populations. Due to space restrictions, we present here details of the six principal CHD policy models, which generated more than one publication that selected for this review (Table 1). Critical appraisals of all 75 papers and all 42 models (including 6 models presented here) are provided in Appendix 3 [see Additional file 1].

### Transparency and limitations of the models

The majority of the models (36, 86%) explicitly stated their key assumptions. However, illustrations or examples of estimations were provided in only 14 (33%). Working versions of the models were potentially available in just 4 (10%).

Barely one fifth of the models reported on limitations of their methodology such as competing causes (8, 19%), lag times (7, 17%) or confounding (8, 19%).

### Papers excluded from the systematic review

Papers excluded are listed alphabetically in Appendix 4 [see Additional file 1] with reasons for exclusion. The commonest reasons for exclusion were that the paper was not a modelling study, that it did not report on CHD outcomes, or that it was only a review.

### Methodology and structure of the six principal CHD policy models

The details on model methodology used in modelling papers included in this review can be seen in the Appendices [see Additional file 1].

The methodology used in models varied widely. ***The Coronary Heart Disease Policy (CHDP) Model ***is a state-transition, cell based model developed in the1980s [13]. It was initially used to examine trends in CHD mortality [14,15] and expected gains in life expectancy from risk factor modifications [16]. This model was also used to evaluate the cost-effectiveness of specific medical interventions for primary and secondary prevention of CHD [7,17-19] and health promotion activities [20].

The model was based on the 1980 US population and mortality statistics. It consists of three sub-models:

- A *demographical/epidemiological *model, which represents the disease-free population aged 35–84 years, stratified by sex, age group and cardiovascular risk factors. This model includes risk factors as categorical variables, therefore over 5,000 cells are required in total. It then uses a logistic risk function based on the Framingham equation to estimate the annual incidence rates of CHD events for each cohort.

- A *bridge model*, which covers subjects for the first 30 days after they develop coronary disease. Using CHD incidence data from Minnesota, the model initially determines whether the first event is angina, myocardial infarction or cardiac arrest[13].

- A *disease history model*, which includes the survivors after the first 30 days, places them in 12 CHD states by age and sex, and then follows them through treatment pathways.

This model allows the user to simulate the effects of an intervention (either risk factor modification, or therapy) by changing case fatality rates and observing the effect on mortality, morbidity and costs for up to 30 years.

***The CHD Policy Analysis Model***, comprised two distinct parts. The primary prevention component of the model developed by the London School of Hygiene and Tropical Medicine aims to simulate the impact of different primary prevention strategies on benefits and costs [21]. The treatment component of the model developed in the Universities of Southampton and Birmingham[21,22] evaluates the impact of different treatments given to two different groups of CHD patients, stable angina or acute myocardial infarction[21].

***PREVENT ***was a cell based model developed by Gunning-Schepers in the Netherlands during the 1980s [23]. It has been used to estimate the health benefits of changes in population risk factor prevalence by comparing i) continuation of existing trends with ii) alteration of the proportions of the population with given risk factor levels. The model allowed one risk factor to be associated with more than one disease and one disease to be associated with more than one risk factor. Demographic evolution was also taken into account in simulations[23].

***The Cardiovascular Life Expectancy Model ***was initially developed by Grover et al in 1992 in Canada to examine the cost-effectiveness of different treatment options for CHD[5]. From 1998 onwards the model was also described as a Markov model. The model includes primary and secondary prevention parts. The *primary CHD part *calculates the annual probability of dying (from CHD or other causes) and the annual risk of CHD events (with or without intervention) for a person without symptomatic CHD at entry to the model. The annual risk of developing specific CHD endpoints is based on data from the Framingham Heart Study.

After developing CHD, a person then moves to the *secondary CHD *model. This part calculates the risk of dying during the 12 months following a non-fatal myocardial infarction. The risk estimations are based on the Framingham logistic equations for primary events after adjustment for the presence of CHD[5].

The difference between the predicted annual cumulative mortality with and without the intervention over the remaining total life expectancy then represents the total years of life saved after that intervention.

***The IMPACT CHD mortality model ***is a cell-based model originally developed by Capewell and colleagues in 1996[6]. Using a MS EXCEL spreadsheet, this model combines data from many sources on patient numbers, treatment uptake, treatment effectiveness, risk factor trends and consequent mortality effects. The ***deaths prevented or postponed ***(DPPs) over a specified time period are then estimated. The model can therefore be used to estimate the proportion of a mortality decline (or increase) over a certain time span that might be attributed to specific treatments or risk factor changes. It can also examine the consequences of increasing treatments provided, or reducing risk factor levels. Other outputs include life years gained and cost-effectiveness of specific interventions (Appendix 3) [see Additional file 1].

***The Global Burden of Disease ***(GBD) model was developed at WHO by Lopez and Murray. It is an example of a model, which uses population attributable risk percentage (PAR %) estimations. The model can calculate the attributable burden of disease for a specific risk factor, population and time, defined as the difference between currently observed burden and the burden that would be observed if past levels of exposure had been equal to the lowest relative risk [24,25]).

The GBD Model has five components: causes of death, descriptive epidemiology of disabling sequelae, burden attributed to selected risk factors, projections of burden for the future and sensitivity analyses. Cause of death data are obtained from vital registrations or other sources. Data on 107 disorders and selected disabling sequel were investigated regarding average age of onset, duration, incidence and prevalence. Burden of disease and injury attributable to ten major risk factors were then calculated. The model uses attributable fractions, taken from reviews and meta-analyses, applied to the population of a region to calculate the burden of disease attributable to these risk factors [25]. Where data were not available, assumed values were used. Burden of disease is measured using disability adjusted life years (DALYs), calculated as the sum of years lost and years lived with disability [26].

### Comprehensiveness

***The Coronary Heart Disease Policy Model ***includes major risk factors such as smoking, total cholesterol, DBP and relative weight, which are necessary to estimate CHD risk using Framingham Equations. The model considers most CHD categories. Individual CHD treatments such as statins, aspirin, and beta-blockers have also been considered in different publications[7,17,18].

The ***PREVENT ***Model is a primary prevention model and therefore only considers risk factors: smoking, cholesterol, hypertension, obesity, physical activity and alcohol use [27-30].

***The Cardiovascular Life Expectancy Model ***estimates the annual risk of developing specific CHD endpoints. Initially based on data published from the Framingham Heart Study from 1998 onwards the model used data from the Lipid Research Clinic follow up cohort. It therefore includes risk factors of age, sex, diastolic blood pressure, total cholesterol, HDL cholesterol level, left ventricular hypertrophy, glucose intolerance and smoking status [31-33]. The only treatment considered was statins. [31-33]

***The CHD Policy Analysis Model ***has separate primary prevention and CHD treatment parts. The primary prevention component includes risk factors such as age, sex, systolic blood pressure, total cholesterol, and smoking[21]. The disease events included are stable angina, unstable angina, myocardial infarction, sudden cardiac death, stroke death, other cardiovascular death, cancer death and death from other known and unknown cause[21]. The CHD Policy Analysis Model originally aimed to include many treatment categories but the only publication thus far focuses on angina [31-33].

***The IMPACT ***Model considers a comprehensive range of risk factors, CHD categories and treatments. For primary prevention the model includes smoking, cholesterol, blood pressure[6,34-36], deprivation, obesity, diabetes, physical activity and also primary prevention with anti-hypertensive medications and statin therapies [37].

The disease categories and treatments include: **AMI**: (Cardiopulmonary resuscitation, thrombolysis, aspirin, angioplasty, beta blockers and ACE inhibitors); **Secondary prevention following MI, and, separately, following CABG or angioplasty**: (aspirin, beta blockers, ACE inhibitors, statins, warfarin and rehabilitation); **Chronic angina**: (CABG surgery, angioplasty, aspirin, statins); **Unstable angina**: (aspirin, heparin, platelet IIB/IIIA inhibitors and clopidogrel); **Heart failure**: (ACE inhibitors, beta blockers, spironolactone, aspirin, statins) and all standard treatments for **Hypertension**.

***The Global Burden of Disease Model ***includes ten major risk factors for global disease burden: malnutrition, poor water quality, unsafe sex, alcohol, occupation, tobacco use, hypertension, physical inactivity, illicit use of drugs, and air pollution[25]. CHD is included in the model, and is modelled as being caused by tobacco use, hypertension and physical inactivity, and reduced by all levels of alcohol consumption.

### Model populations

Most of the models were restricted to young and middle-aged groups, generally 15 to 64 years (Tables 1–7 in Appendix 3) [see Additional file 1]. However, the CHD Policy Model, IMPACT and the CHD Policy Analysis Model considered groups aged up to 84 years. None of the models specifically considered non-Caucasian populations.

### Model outcomes

The most common outcomes reported were numbers of deaths prevented, life-years gained, CHD incidence and cost or cost-effectiveness (Table 1 and Appendix3) [see Additional file 1]. Among the six models included in this paper, CHD Policy Model, PREVENT, IMPACT, Global Burden of Disease and CHD Policy Analysis have capability to predict future trends in disease and other related outcomes except the Cardiovascular Life Expectancy Model. This model has been used to assess cost-effectiveness of interventions rather than predicting or explaining the trends.

### Model quality

Relatively few papers included in this review reported on quality issues. In a few models, sensitivity analyses were reported, examining the effect of varying key factor such as treatment costs or measurement imprecision. However, the majority were one-way analyses (varying one factor at a time), rather than the more rigorous multi-way sensitivity analyses (varying several factors simultaneously) (Table 1 and Appendix 3) [see Additional file 1].

Assessment of validity was reported in very few models. In the CHD Health Policy Model, this was done by comparing the CHD deaths estimated by the *model *with the *actual *CHD deaths observed in 1990 [14]. In the IMPACT Model, validity was likewise checked by comparing the *estimated *fall in CHD deaths with the *observed *fall in specific age and sex categories[6,37]. In PREVENT, model validity was checked by comparing model estimates with another estimation method[38]. In the Cardiovascular Life Expectancy Model, predictive validity was checked by comparing the model estimates with events observed in primary and secondary prevention trials[39,40]. The original 1992 Cardiovascular Life Expectancy model was similarly validated against the data set from the Lipid Research Clinic cohort [32].

The CHD Health Policy model was similarly calibrated using life years estimated from the model compared with life expectancy from 1980 national statistics [16]. Only two of the models had been replicated in different populations (PREVENT[28,41] and IMPACT [6,10,34-37,42,43]).

## Discussion

This is the first comprehensive systematic review of CHD policy models. Previous reviews were restricted to a particular type or application [44-46]. The increasingly wide use of CHD modelling has thus far resulted in few attempts to evaluate model quality. We therefore aimed to systematically assess the quality of the modelling methodology rather than simply comment on the reported results.

A wide variety of CHD policy models have been developed with over 70 publications now available. CHD models have become more complex and comprehensive as a result of improving computer technology and wider usage[10]. Although, on the surface many of the models may appear similar, one must remember that they were often originally designed to address very different policy questions.

In general, the quality of the models has also improved over time so that more recent papers tend to explicitly report assumptions, limitations and sensitivity analyses.

Quality criteria assessing for publications are well described, especially for randomised controlled studies[47]. However, there are no widely accepted quality criteria for modelling papers in general nor specifically for CHD policy models. We therefore first had to develop an evaluation framework using logical quality criteria including sensitivity analyses, validity, and comprehensive reporting of assumptions and limitations (Appendix 3) [see Additional file 1]. These criteria explicitly reflect the main quality components suggested in the recent ISPOR Guideline [3].

Models can allow a large amount of evidence to be considered simultaneously, by combining and integrating into a coherent whole different types of data from controlled trials, routine surveillance and expert consensus[10]. Models have been extensively used in policy making and resource allocation, since they permit policy makers to examine future options, or to simulate the effects of different scenarios within a population [4]. However, improved technology potentially increasingly enables practitioners and policy-makers to use these models without necessarily understanding the inherent assumptions or data limitations [10].

Models require considerable data input. The data sources therefore need to be appropriate and credible. However, the availability of comprehensive high quality data remains a problem. The data are therefore usually obtained from a variety of sources including clinical trials, meta-analyses, surveys, clinical databases and registries, medical records, audits, routine statistics, official tariff lists for health care resource use and Delphi panels (to elicit expert opinion)[48]. Every CHD modelling paper should therefore explicitly report and critically discuss data quality, methodological limitations and the assumptions used to address these deficiencies. However, few of the papers reviewed here did so.

Uncertainties about data are a perennial problem in modelling studies. Sensitivity analyses are therefore essential to quantify the degree of uncertainty. In general, CHD models have only recently started to report sensitivity analyses [14,40,49]. The most common approach is where one or more parameters of an evaluation are varied across a plausible range[50]; 95% confidence intervals can easily be included. ***One-way sensitivity analysis ***(where only one parameter is changed at a time while the others retain their base-case specifications) is obviously less rigorous than ***multiway-sensitivity analysis ***(where more than one parameter is changed at the same time). However, multiway sensitivity analyses remain very uncommon [6,10,37]. Lately, the use of **probabilistic sensitivity analysis **has been suggested alongside with traditional sensitivity analyses. Probabilistic sensitivity analyses offer the opportunity to make statistical statements about the impact of parameter uncertainty in models. Although, this approach has been criticized for its arbitrariness in choosing the statistical distribution it may be used more often in the future [51]. Probabilistic sensitivity analysis was not used in any of the reviewed models in this paper.

### Implications for policy and practice

Many of the papers included in this systematic review failed to provide sufficient detail to allow thorough evaluation of the model used. This obviously constrained our systematic review. When assessing the quality of a model, one should ideally consider the system being modelled, the model structure, the elements included and excluded, the probable effects of existing trends in mortality and risk factors and the model assumptions-both explicit and implicit[3,4]. The description of the model should be sufficiently detailed so that the model can be replicated mathematically. Therefore modeling papers should include some basic information on models listed in Table 2. If the validity of a model cannot be checked, it simply represents a "black box" rather than a useful tool. More extensive validation studies thus represent a priority for future research. Although most of the models have potential to predict future trends depending on how the model is populated, the methodological challenges are considerable, and there is substantial need for further research and development.

Finally, although this review focussed on CHD models, many of the issues are generic. Some findings may therefore be cautiously generalisable when modelling other diseases or interventions.

## Conclusion

In conclusion, CHD models offer a potentially valuable tool for policy development. However, existing models vary widely in their depth, breadth and quality. Few models have been calibrated, replicated or validated against minimum quality criteria. Before being accepted as a policy aid, any model should explicitly include a statement of its aims, assumptions, outputs, strengths and limitations.

## Abbreviations

CHD-Coronary heart disease

AMI-Acute myocardial infarction

CABG-Coronary artery bypass graft

MI-Myocardial infarction

LYG-Life years gained

PTCA-Percutaneous transluminal coronary angioplasty

## Competing interests

Belgin Unal was funded by the NHS Executive North West Research and Development Directorate as a Research Training Fellow.

All other authors declare that they have no competing interests.

## Authors' contributions

BU developed the review protocol, searched the electronic databases, selected the papers by applying the inclusion and exclusion criteria, critically appraised the selected papers, summarised the findings and interpreted the results, drafted and wrote the paper.

SC contributed to the conception and design of the study, revised the review protocol, selected the papers by applying the inclusion and exclusion criteria, critically appraised the selected papers, interpreted the results, revised and contributed to the paper.

JC contributed to the conception and design of the review. Critically reviewed the findings and interpreted the results and revised the paper.

All authors read and approved the final manuscript.

## Pre-publication history

The pre-publication history for this paper can be accessed here:



## Supplementary Material

Additional File 1 Appendices for CHD policy models review. The file presents search strategy, quality review of CHD models, summary tables and the list of excluded studies. Click here for file

## Figures and Tables

**Table 1:** Summary of the six principal CHD policy models

**Name of the model (Author)**	**Type of model**	**Model setting & Study population(s)**	**Risk factors included**	**Disease groups & treatments included**	**Outcomes**	**Sensitivity analysis**	**Validation**	**Strengths and limitations**
**CHD Policy Model (Weinstein and Goldman)**	State transition Markov Model	USA, Men and Women aged 35–84	Smoking, total cholesterol, DBP and weight to estimate CHD risk using Framingham Equations	Angina, AMI, sudden death, post MI, CABG, PTCA Specific treatments considered in different studies eg statins, aspirin, beta-blockers etc	Number of deaths prevented, LYG, CHD incidence (number of arrests, angina, AMI), CHD prevalence, CHD mortality, cost per life year	In the initial model none. Subsequently papers reported one way sensitivity analysis	Model was calibrated using 1986 mortality data. Validity: Model estimates were compared with 1990 observed-92–98% fit reported.	First policy model rather basic. Steadily refined since then. Many papers in high impact journals
**PREVENT (Gunning-Scheppers)**	Cell based	Netherlands; Denmark, England Depending on the purpose aged <65	Smoking, cholesterol, hypertension, obesity, physical activity, alcohol	None	Number of deaths prevented, life years gained	One way, different scenarios	Not checked	Mainly a primary prevention model. Developed and adopted in several different populations.
**CHD Life Expectancy Model (Grover et al)**	Life table analysis-Markov model from 1998 onwards	Canada, Adult men and women, age group not clear	Smoking, total cholesterol, DBP, glucose intolerance, age	Did not consider CHD disease categories but treatments can be considered for primary prevention	Years of life saved, cost per life year saved, years of life without CHD symptoms	One-way	Calibrated	This model uses hypothetical cohorts of participants. In most of the papers, time and the specific population are not clear.
**CHD Policy Analysis (Sanderson and Davies)**	Micro simulation	England and Wales, Up to 85 years. Men and women	Smoking, cholesterol, systolic blood pressure	Angina (stable and unstable), AMI, postMI, CABG, PTCA None	Deaths prevented, morbidity prevented, CHD & non-cardiac deaths, unstable angina admissions, investigations, angiograms, PTCA, CABG		No validation reported	Separate risk factor and treatment components. Future model may include secondary prevention treatments. No sensitivity analyses yet. Model fit appears better for men than women.
**IMPACT (Capewell, Critchley and Unal)**	Spread-sheet	Scotland, England & Wales, New Zealand. Initially men and women aged 45–84. IMPACT Model for England and Wales includes 25–84	Initially smoking, cholesterol, blood pressure – then also obesity, diabetes and physical activity and deprivation	This model is comprehensive and considers all principal CHD categories and over 20 specific CHD treatments	Deaths prevented or postponed, life years gained.	Multi way sensitivity analysis using Analysis of extremes method.	Estimated falls in CHD mortality were compared with observed falls over specific time period stratified by age and sex.	Considers all major effective treatments available for CHD and all major risk factors. Data quality adequate, used trial and meta-analyses: National population statistics and results from representative studies
**Global Burden of Disease (Murray and Lopez)**	Population attributable risk method	World divided into eight geographic regions M-F all ages	Malnutrition, poor water, unsafe sex, alcohol, tobacco occupation, hypertension, physical activity, illicit drugs, and air pollution	None	Disability adjusted life years (DALYs)	Multi-way sensitivity analysis-discounting and age weighting	None	A comprehensive and global model for WHO strategies. Well documented and described. CHD is included, and modelled as caused by tobacco use, hypertension and physical inactivity, and reduced by alcohol. Data quality: Extremely variable depending on the region

**Table 2:** Suggestions for future papers reporting on CHD policy models

**A CHD modelling paper should explicitly report and discuss;**
-Aims of the project
-Structure and methods of the model
-Data quality (data availability, how up to date, comprehensive, any gaps in certain population groups or interventions). Reasons for selecting or excluding specific data sources
-Methodological limitations -The assumptions used to address these deficiencies
-Sensitivity analyses (one-way or preferably multi-way)
-Whether the validity of the model was checked (with real observational data or with other models)
-Replication of the model in different populations
-Model results and comparisons with other studies
-Social and economic policy implications of model outcomes
Suggestions for future research
