# Supplementary material for: Coronary heart disease policy models: a systematic review
Source: BMC Public Health. 2006 Aug 18;6:213. doi: 10.1186/1471-2458-6-213 (PMC1560128; doi:10.1186/1471-2458-6-213)
Supplement: Additional File 1 — Appendices for CHD policy models review. The file presents search strategy, quality review of CHD models, summary tables and the list of excluded studies. [file 1471-2458-6-213-S1.doc]

# APPENDICES FOR CHD POLICY MODELS REVIEW

**CONTENTS**

| APPENDICES | Page Number |
| --- | --- |
| Appendix 1. Search strategy for CHD policy models review | 2 |
| Appendix 2. Quality Review of CHD Models IN/OUT FORM and data extraction form | 3 |
| Appendix 3. Summary tables for systematic review of CHD policy models | 8 |
| Appendix 4. List of excluded studies | 40 |
| References | 41 |

# Appendix 1. Search strategy for CHD policy models review

1. exp Cardiovascular Diseases/

2. (coronary adj10 (disease$ or event$ or atherosclero$ or arteriosclero$ or thromb$)).tw.

3. (heart adj10 (attack$ or isch?emi$ or arrest or disease$)).tw.

4. (myocardial adj10 (infarct$ or isch?emi$)).tw.

5. angina$.tw.

6. (CHD or IHD or CAD).mp.

7. (CHD or IHD or CAD).tw.

8.(sudden$ adj10 cardiac).tw.

9. 1 or 2 or 3 or 4 or 5 or 6 or 7 or 8

10. coronary.tw.

11. exp Myocardial Infarction/

12. exp Coronary Disease/

13. exp Coronary Arteriosclerosis/

14. *Arteriosclerosis/

15. exp Arteriosclerosis Obliterans/

16. exp Coronary Thrombosis/

17. exp Myocardial Ischemia/

18. Heart Failure.tw.

19. 9 or 10 or 11 or 12 or 13 or 14 or 15 or 16 or 17 or 18

20. model$.tw.

21. simulat$.tw.

22. prevent$.tw.

23. treat$.tw.

24. 20 or 21

25. 22 or 23

26. 19 and 24 and 25

27. POHEM.tw.

28. CRISPERS.tw.

29. exp Decision Making/ or decision making.mp.

30. exp Health Care Policy/ or health policy.mp.

31. exp Public Health/ or public health.mp.

32. 26 or 27 or 28

33. 29 or 30 or 31

34. 32 and 33

35. limit 34 to human

# Appendix 2. Quality Review of CHD Models IN/OUT FORM and data extraction form

**Paper:** (Author, Year)

**Ref ID:**

#### **Reviewer: Date:**

|  | *Please tick one of the options* | | |
| --- | --- | --- | --- |
|  | **Yes** | **No** | **Unclear** |
| 1. **Do the study refer to modelling*?** |  |  |  |
| **2- Does the study deal with the population rather than the individual?** |  |  |  |

| 1. **Does the study report on one or more of the following health outcomes?**   ***(Please tick)*** | | |
| --- | --- | --- |
| CHD deaths prevented |  |  |
| CHD disease prevented |  |  |
| CHD mortality |  |  |
| CHD prevalence |  |  |
| CHD incidence |  |  |
| CHD prevention OR treatment cost |  |  |
| Life years gained |  |  |
| Disability |  |  |
| Hospital admission for CHD |  |  |

**Final decision about a paper:**

| *Tick* |  |  |
| --- | --- | --- |
|  | **‘In’** | - ‘yes’ to questions 1-2 **and** - includes at least one of the outcomes in question 4 |
|  | **‘Pending’** | if any of the sections are ‘unclear’ |
|  | **‘Out’,** | if any of the sections are ‘no’ ** |

*: for the purpose of this review, modelling is defined as attempts to create tools which help predicting outcome of interventions or explain observed trends (by risk factor change or specific treatment effect or implementation a new strategy) on population level.

**: If 2 reviewers disagree, they can then discuss

# DATA EXTRACTION FORM for CHD Models Review

**Paper:** (Author, Year)

**Ref ID:**

# Reviewer: Date:

# A) MODEL DETAILS

**Name of the Model:** ………………….

**Author of the model:** ………………….

**Purpose of the model:** ………………….

### Model Setting

Country / Area:

### Study Population

General description of population (Age, sex structure)

**Time period which the model covers**

Baseline modelling period:

Prediction period:

| **Type of model** | Please tick every method |
| --- | --- |
| Simulation |  |
| Micro simulation |  |
| Spread sheet |  |
| Life table analysis |  |
| Markov model |  |
| Monte-Carlo model |  |
| Others ………. | |

| **Model Description:** |
| --- |

| **RISK FACTORS INCLUDED** | **Tick please** | **Form*** | **Intervention *(describe)*** |
| --- | --- | --- | --- |
| Primary prevention |  |  |  |
| Smoking |  |  |  |
| Cholesterol |  |  |  |
| HDL-C |  |  |  |
| LDL-C |  |  |  |
| Triglycerides |  |  |  |
| Drug therapy (ie statins for prim prev?) |  |  |  |
| Blood pressure DBP/SBP |  |  |  |
| Diabetes |  |  |  |
| Physical activity |  |  |  |
| Deprivation |  |  |  |
| Obesity or BMI |  |  |  |
| Diet-nutrition |  |  |  |
| Other |  |  |  |
| Secondary prevention*(Please specify the TX)* |  |  |  |
| Tx1: …… |  |  |  |
| Tx2: …… |  |  |  |
| Tx3: …… |  |  |  |
| Tx4: …… |  |  |  |
|  |  |  |  |
|  |  |  |  |
| Rehabilitation |  |  |  |
| Smoking cessation |  |  |  |
| Diet |  |  |  |
| Other |  |  |  |

| **Disease categories included** | | *Please tick*  * If the variable was modelled as continuous use ‘Con’  **if it was categorical use ‘Cat’** | |  | |
| --- | --- | --- | --- | --- | --- |
| Angina | |  | |  | |
| AMI | |  | |  | |
| Sudden cardiac death/Arrest | |  | |  | |
| Post MI | |  | |  | |
| Heart failure | |  | |  | |
| CABG | |  | |  | |
| PTCA | |  | |  | |
| **DATA SOURCES USED:** | **Source** | | **Comments on quality**  (Please consider sample size and response rates for surveys/ national data etc) | | **Limitations** |
| Population data |  | |  | |  |
| Mortality number/rate |  | |  | |  |
| Morbidity number/rate |  | |  | |  |
| Treatment uptake |  | |  | |  |
| Risk factor  Prevalence/ trends |  | |  | |  |
| Treatment effectiveness |  | |  | |  |
| Risk factor change effectiveness/ Betas |  | |  | |  |
| Others |  | |  | |  |

**TYPE OF OUTCOMES STUDIED *Tick please***

| Number of deaths prevented |  |
| --- | --- |
| Number of morbidity (MI/ HF/ etc?) prevented |  |
| CHD mortality |  |
| Prevalence |  |
| Incidence |  |
| Cost (per life year, per death prevented..) |  |
| Life years gained |  |
| Hospital admission for CHD |  |
| Others (please describe) |  |

**Please describe ‘main’ outcome of the study in the author’s words:**

| **SENSITIVITY ANALYSIS** | | No | | Yes | |
| --- | --- | --- | --- | --- | --- |
| Any sensitivity analyses carried out? | |  | |  | |
| Were 95% CIs for RRs used for sensitivity analyses? | |  | |  | |
| Which sensitivity analyses were carried out? *(Analysis of extremes, One- Multi way, other?)*  ………………………………………………………………………………… | | | | | |
|  | Poor | | Reasonable | | Good |
| Were sensitivity analyses discussed? |  | |  | |  |

| **CALIBRATION** | No | Yes |
| --- | --- | --- |
| Was the model calibrated? |  |  |
| How was the model calibrated? ***Describe……..*** | | |

| **PREDICTIVE VALIDITY** | No | Yes |
| --- | --- | --- |
| Was the validity of the model tested? |  |  |
| How was the validity of the model checked? ***Describe……..*** | | |
| How was the validity quantified? *(eg % explained)……………..* | | |

| **TRANSPARENCY** | Not available | Yes (Available) |
| --- | --- | --- |
| Illustrations/ examples |  |  |
| Assumptions |  |  |
| Model availability for reader |  |  |

| **POTENTIAL LIMITATIONS** | Not Reported | Reported | Discussed | Method refined |
| --- | --- | --- | --- | --- |
| Assumptions |  |  |  |  |
| Confounding |  |  |  |  |
| Lag times |  |  |  |  |
| Competing causes |  |  |  |  |

**Other comments on the study:**

# Appendix 3. Summary tables for systematic review of CHD policy models

Table 1. Summary table for CHD Policy Model papers

| **Author (year)** | **Purpose of the model** | **Setting, Time period & Population** | **Risk factors & interventions included** | **Disease categories & Treatments included** | **Outcomes** | **Key Results** | **Sensitivity analysis or validation** | **Transparency** | **Data Quality & Strengths and Limitations** |
| --- | --- | --- | --- | --- | --- | --- | --- | --- | --- |
| **Goldman 1984^[1]^** | To analyse potential effect of medical interventions and changes in life style | USA, 1968-1976, general USA population | Smoking, cholesterol, blood pressure, obesity | AMI,  Angina, Sudden death, post MI  β Blockers, antihypertensive, CABG surgery | Number of deaths prevented | More than half the CHD decline 1968-1976 was related to changes in lifestyle (cholesterol, smoking).  About 40% could be directly attributable to specific medical interventions, mainly CCU, medical treatments of CHD and hypertension | None | Assumptions discussed.    Model not available.  No illustrations, no discussion of confounding, lag times or competing causes. | Data sources adequate. The first published attempt to explain CHD mortality fall in terms of treatments and risk factor changes. Clear estimates and assumptions. Admits limitations. |
| **Weinstein 1987^[2]^** | To project future mortality, morbidity, and cost of CHD | USA, 1980-2010 M-F, aged 35-85 | Smoking, cholesterol, blood pressure, obesity (relative weight) | Angina, AMI, Sudden death, CABG    No specific treatments included | Number and rate of  - CHD events  (Arrest, angina, AMI)  - CHD deaths  - CHD prevalence, incidence  - Resource cost of interventions  -All cause mortality | Predicted by 2010:  10% decline in CHD incidence rates  38% increase in CHD events  50% prevalence increase  46% increase in deaths | None | Assumptions discussed.  Model not available.  No illustrations, nor discussion of confounding, lag times or competing causes. | First Policy Model, rather basic, steadily refined since then.  Data sources adequate. |
| **Goldman 1989^[3]^** | To evaluate long term national effects of lowering cholesterol | USA, 1990-2015 all 35-84, M-F | Smoking, cholesterol, blood pressure, obesity (relative weight)  Interventions:  1-To reduce high cholesterol (>250 mg/d) to 250 mg/dl in all people, in 1990.  2- population wide cholesterol reductions to reach the same benefit | No disease categories  No specific treatments included | Number of disease cases prevented | Targeted programme would reduce CHD incidence by 8-10% in men 35-54 and by 1-4% in men 55-74.  10mg/dl reduction in men pop mean cholesterol and 23% red in women pop would achieve similar result.  Relying on targeted cholesterol reduction would be inadvisable to reduce national CHD. | One-Way sensitivity analysis  Model was calibrated but predictive validity notchecked | Illustrations: Yes  Assumptions discussed.  Model not available. Confounding & lag times discussed, but not competing causes | Data sources adequate. |
| **Tsevat 1991^[4]^** | To determine potential gains in life expectancy from risk factor modifications | USA, 1990, people who turned to 35 in 1990 | Smoking, cholesterol, dbp, relative weight | No disease categories included  No treatments included | Gains in life expectancy | Pop gain in life expectancy (Years in Men/ Women) IF);   1. 10mg/l red in cholesterol- (0.2-0.2 years); 20 mg/l (0.4-0.3yr), all high cholesterol reduced to 240mg/dl (0.3-0.5yr) 2. smok halved (0.4-0.4 yr) 3. dbp below 88 (1.1-1.1 yr) 4. weight ideal (0.6-0.4 yr) 5. eliminate all CVD (3.1-3.3 yr) | One-Way sensitivity analysis  Calibrated- life years estimated compared with US life expectancy from 1980 national vital statistics | Model not available. No illustrations.  Discussed assumptions, lag times & competing causes but not confounding | Risk factors assumed to be independent therefore coefficients might cause underestimation.  Data sources adequate. |
| **Goldman 1991[5]** | To determine cost effectiveness of HMG-CoA reductase inhibitor in primary and secondary prevention | USA, 1989, 35-84 M-F | Smoking, cholesterol, dbp, relative weight | No other treatments included | Cost per life year saved | Lovastatin 20 mg/d save lives and costs in young men with cholesterol >250mg/dl and have favourable cost effectiveness ratio regardless of cholesterol level except in young women with cholesterol<250mg/dl. Doses of 40 mg/dl had favourable cost effectiveness ratio in men with cholesterol>250mg/dl. By comparison primary prevention with lovastatin had favourable cost effectiveness ratio only in selected groups based on cholesterol levels and other established risk factors. | Different scenarios examined.  Calibrated- life years estimated compared with US life expectancy from 1980 national vital statistics | Model not available. No illustrations.  Discussed assumptions, lag times & competing causes but not confounding | Restricted focus  Data sources adequate. |
| **Hunink 1997[6]** | To examine effect of secular trends in risk factor levels and improvements in treatments on CHD mortality decline in USA, 1980-1990. | USA, 1980-1990 35-84 M-F | Smoking, cholesterol, hdl, ldl, dbp | Angina, sudden death, post MI, CABG, PTCA  Specific treatments not included | Number of deaths prevented | Model explained 92% of the observed decline.  43% of the fall was attributed to treatments and 25% to primary prevention | One-way sensitivity analysis- 95%CIs from case fatalities and Beta coefs.  Model calibrated with 1986 mortality data-  98% Validity: (model estimates compared with 1990 observed)- | No illustrations, model is not available. Assumptions discussed and method refined.  Discussed Confounding, lag times & competing causes. | Data sources well explained- However model excluded over 85 people, did not consider specific treatments and excluded heart failure. |
| **Tosteson 1997[7]** | To estimate cost effectiveness of population wide approaches to reduce cholesterol in US adult pop. | USA, 1995-2020, 35-84 M-F, free of CHD | Smoking, cholesterol, hdl, dbp | No disease categories included  No treatments included | CHD incidence, lyg, cost per lyg, CE ratio | A population wide programme with the cost (4.95 per person per year) and cholesterol lowering effects (an avr. 2% reduction) would prolong life at an estimated cost of $3200 per life year saved. | One-Way sensitivity analysis  Model calibrated (in previous papers), predictive validity –not checked | No Illustrations  Model not available  Discussed competing causes but not assumptions, confounding or lag times | Data sources adequate |
| **Goldman 1999[8]** | To project the population wide effect of full implementation of ATP II guidelines in the USA | USA, 2000-2020, 35-84 M-F | Smoking, cholesterol, HDL, LDL, DBP  Primary /secondary prevention in high risk persons and primary prevention in moderate risk persons. | Angina, AMI, sudden death, post MI, CABG, PTCA  No treatments included | Number of deaths prevented, lyg, QALY | ATP implementation means 500 million person years on lipid lowering treatment (2/3 primary prevention and 1/3 on secondary prevention) with 2 million fewer AMIs, 1.7 million fewer CHD deaths, PLUS 14 million LYGs and 13.5 million QALYS | One-Way sensitivity analysis  In previous papers model calibrated  Predictive validity –not checked | Assumptions presented.  Model not available, no illustrations.  No discussion of confounding, lag times or competing causes | Data sources adequate |
| **Phillips 2000[9]** | To examine the potential health and economic impact of increase use of beta-blockers in AMI survivors | USA, 2000-2020;  AMI Survivors in 2000 aged 35-84, followed up for 20 years  PLUS successive survivors of first MI from 2000 to 2020 | None | Post MI  Treatment: beta blocker use after AMI | Number of deaths prevented, cost per life years, QALY | Increase of beta-blocker uptake from 44% to 92%.  Implementing this strategy in MI survivors in 2000: would lead to 4,300 fewer CHD deaths and 3,500 AMIs PLUS 45000 LYGs. Cost per QALY=$4,500.  All first MI survivors annually over 20 years: 72,000 fewer CHD deaths, 62,000 fewer AMIs PLUS 447,000 LYGs. Would save $118 million during. 20 years | One way sensitivity analysis- different scenarios were explored  No validation or calibration in here | No Illustrations. Model not available.  Discussed assumptions but not confounding, lag times or competing causes. | Model described well but the purpose was very narrow  Data sources adequate |
| **Prosser 2000[10]** | To evaluate cost effectiveness of primary and secondary prevention with cholesterol lowering drugs in separate risk groups | USA, from 1987 for 30 years  M-F aged 35-84 | HDL, LDL | Angina, post MI  Treatment: Statins  Step 1 diet | Number of deaths prevented, QALY, cost effectiveness ratio | Cost per QALY for step 1 diet generally <$100 k if subjects had more than 1 RF.  Primary prevention with statins expensive  varied $54k- 240 k in men,  $62 k to 1400 k in women.  Secondary prevention with statins $3800- $9900 per QALY in men and $8100-4000 per QALY in women. | One-Way and multi-way sensitivity analysis done  Calibrated and predictive validity checked | No Illustrations provided. Model not available.  Discussed assumptions, but not confounding, lag times or competing causes. | Useful.  Data sources adequately reported |
| **Goldman 2001[11]** | To estimate impact and cost effectiveness of risk factor reductions between 1981 and 1990. | USA, 1981-1990 and 1991-2015; 35-84 M-F | Smoking, cholesterol, dbp, obesity | Angina, AMI, sudden death, pot mi, CABG, PTCA  No treatments included | Number of deaths prevented, incidence, cost per death prevented | RF changes between 1981-1990 resulted in 7-11% reduction in CHD incidence rates- 430,000 fewer CHD deaths. 55% of this reduction was from dbp, 38% cholesterol, 7% smoking.  Overall RF changes gained 1.9 million QALYs | One-Way sensitivity analysis  Calibrated- in other papers | No Illustrations. Model not available.  Discussed assumptions, lag times & competing causes but not confounding | Ambitious paper, difficult to understand and cost estimations slightly confusing.  Data sources adequately reported |
| **Tice 2001[12]** | To examine the potential effect of grain fortification with folic acid and  vitamin therapy i.e. cyanocobalamine, on CHD events in the US. | USA, 2001-2010; 35-84 M-F | Smoking, cholesterol, hdl, dbp, diet (folic acid fortification) | Angina, AMI, heart failure, QALYs  No other treatments included | Number of deaths prevented, number of CHD events prevented, QALYs | Grain fortification would decrease AMI in men and women by 13% and 8% respectively.  310, 000 fewer deaths and lower costs if all known CHD patients treated with folic acid and cyanocobalamin over 10 years  Providing all men over 45 without CHD would save 300,000 QALYs and would save $2 billion | Multi-Way sensitivity analysis  By incorporating homocysteine level distribution from NHANES III.  This version of the model apparently predicts CHD mortality within 2% of the 1990 US vital statistics. | No illustrations and model not available.  Discussed assumptions, Lag times & competing causes but not confounding | Assumed 100% compliance- Same RR assumed for primary and secondary prevention- no negative effect of rx, - lack of completed RCT evidence  Data sources adequately reported |
| **Gaspoz 2003[13]** | To estimate cost effectiveness of aspirin, clopidogrel or both for secondary prevention | USA, 2003-2027; 35-84 M-F | No risk factors included | Two treatments for secondary prevention of CHD Aspirin, Clopidogrel | Number of deaths prevented, cost per lyg and QALY | Cost per QALY results:    Aspirin for all eligible patients =$11,000-  Aspirin all and clopid for others: =$31,000-  Clopidogrel for all  =$250,000 | One-Way sensitivity analysis  Using cholesterol changes in 4S Study the model estimated almost perfectly the observed CHD events in the trial. | No illustrations, model not available.    Discussed assumptions, lag times & competing causes but NOT confounding or compliance | Narrow focus.  Data sources adequately reported |

# Table 2. Summary table for PREVENT Model

| **Author (year)** | **Purpose of the model** | **Model setting, Time period & Population** | **Risk factors included** | **Disease categories & Treatments included** | **Outcomes studied** | **Key Results** | **Sensitivity analysis & Validity** | **Transparency** | **Strengths / Limitations &Data Quality** |
| --- | --- | --- | --- | --- | --- | --- | --- | --- | --- |
| **Buck, 1996[14]** | To simulate the health outcomes associated with health promotion and prevention and relative costs of associated with different health promotion | England, 2000-2029, M-F under 65 | Smoking, cholesterol, blood pressure | None | Number of deaths prevented, cost | Reducing smoking by 2.5% over 1 yr in the population; would reduce CHD death numbers by 2,378 in 2000, 31,602 in 2029. Reducing cholesterol 5% over 3 yrs; would avert 67,598 CHD deaths in 2000, 438,396 CHD deaths in 2029 | Different scenarios explored  Validity not checked | Illustrations& assumptions provided.  Model not available.  Discussed confounding & competing causes but not lag times. | Ignore socioeconomic variables  Data sources adequate |
| **Naidoo, 1997[15]** | To evaluate effect of physical activity in reducing CHD deaths | England and Wales, Base year: 1991  Intervention 1994-2005 then 14 years prediction (to 2019),  M-F aged 15-64 | Smoking, cholesterol, blood pressure, physical activity, obesity | No treatments | Number of deaths prevented, LYG | Increasing physical activity would result in small reduction in CHD death rates (0.15% in men & 0.06% in women). Greatest health gain can be achieved by concentrating on sedentary people, on older people and on men  Much bigger potential gains from smoking reduction | One-Way sensitivity analysis  Validity not checked | Illustrations & model not available  Discussed assumptions, lag times, competing causes but not confounding. | Physical activity included here in the PREVENT Model. Assumed complete reversal of prior risk from being sedentary.  Data adequate, RR: 1.9 from Berlin et al still reflects cohorts not interventions, probably a big overestimation. |
| **Bronnum-Hansen, 2000[16]** | To estimate smoking attributable mortality from lung cancer, chronic bronchitis, emphysema, CHD, and stroke, by using PREVENT Model and the method proposed by Peto et al. | Denmark, 1993, M-F | Not reported | None | CHD mortality | In 1993 PREVENT model estimated 33% of the deaths in men and 23% in women could be attributable to smoking. The Peto method estimated 35% of deaths in men and 25% in women attributable to smoking. | No sensitivity analysis  Validity thus checked | Illustrations & model not available  Discussed assumptions, lag times, competing causes but not confounding. | Data sources poorly reported and discussed |
| **Mooy, 2000[17]** | To evaluate three policy options (anti tobacco, cycling, high fruit-vegetable consumption) using PREVENT Model | Netherlands, 1993-2003,  M-F under 65 | Smoking, physical activity (cycling), diet | None | Life years gained (LYG) | Anti-tobacco policy had greatest impact, a cycling policy resulted in substantial health gain, increased fruit-vegetable consumption had little effect | Different scenarios explored  Validity not checked | Illustrations & assumptions provided.  Model not available.  Discussed lag times but not confounding or competing causes. | Superficial paper lacks detail. Rather brave and optimistic assumptions??  Data sources reported poorly |
| **Bronnum-Hansen, 2002[18]** | To predict effect of reducing prevalence of hypertension, high cholesterol, smoking and increasing physical activity | Denmark, 1999-2008,  M-F aged 20-64 | Smoking, cholesterol, hypertension, physical activity | None included | Number of deaths prevented | Reducing smoking by 1/3 over 10 yrs would reduce CHD deaths 10% for men and 15% for women < 65.  If heavy smokers or hypertensive reduced by 25% the CHD mortality would be 5% lower for men (6-7% lower women.)  Reducing number with cholesterol (>8mmol/l) by 25% would lower CHD mortality by 3% in men (6% in women) after 15 yrs. | One-Way sensitivity analysis  Validity not checked | Illustrations & model not available    Discussed assumptions & competing causes but not lag times or confounding. | Data sources reported poorly |

# Table 3. Summary table for Cardiovascular Life Expectancy Model

| **Author (year)** | **Purpose of the model** | **Model setting, Time period & Population** | **Risk factors included** | **Disease categories & Treatments included** | **Outcomes studied** | **Key Results** | **Sensitivity analysis & Validity** | **Transparency** | **Strengths / Limitations &Data Quality** |
| --- | --- | --- | --- | --- | --- | --- | --- | --- | --- |
| **Grover, 1992[19]** | To evaluate life-time benefits of reducing total cholesterol levels to prevent CHD | Canada,  (time not clear), Low risk and high risk M-F | Smoking, total cholesterol, diastolic blood pressure, glucose intolerance, age | No disease categories  No other treatments | Years of life saved,  Years of life without CHD symptoms  Increased life expectancy | In low risk and high risk men and women reducing serum cholesterol levels by 5% to 33% would increase the average life expectancy by 0.03 - 3.16 years. Onset of symptomatic CHD would be delayed among these patient groups by 0.06 - 4.98 years, on average | None  Validation only checked for middle age men  Not clear about model fit for other age and sex groups. | Illustrations &: assumptions provided  Model not available.  Discussed lag times but not confounding or competing causes | Fairly clear &, detailed paper.  This model used multivariate logistic regression coefficients.  Data sources adequate |
| **Hamilton, 1995[20]** | To evaluate life-time cost effectiveness of statins for treating high cholesterol levels | Canada,1993  (not clear) M-F aged 30-70 free of CHD | Smoking, total cholesterol, HDL-C, LDL-C, diastolic blood pressure, glucose intolerance | No disease categories  No other treatments | Years of life saved,  Cost per life years saved  (lys) | Treatment of hypercholesterolemia relatively cost-effective for men (as low as $20,882 per LYS at age 50) & women ($36,627 per LYS at age 60). | One-Way sensitivity analysis  Validation reported in previous papers | Illustrations& model not available.  Assumptions& competing causes discussed but not confounding or lag times. | Assumed 100% compliance to treatment (!).  Treatment effectiveness data from only one RCT, no meta-analysis. Data sources adequate |
| **Grover, 1998[21]** | To compare potential years of life saved associated with risk factor modification in the primary and secondary prevention of CVD | Canada, (year not clear)  Lipid Research Clinics Cohort over 30 M-F | 1992 paper  not clear | No disease categories  No other treatments | Number of CHD, stroke cases prevented, life years saved (LYS) | In hyperlipidemic men and women without CVD lipid therapy benefits greater in high-risk vs low-risk groups (4.74-0.78 vs 2.50-0.25 LYS, respectively).  Similar forecasted benefits among people with CVD.  Hypertension therapy benefits also greater for high-risk vs low risk (1.34-0.29 vs. 0.85-0.13 LYS respectively) | No sensitivity analysis  Predictive validity checked by using data from primary and secondary prevention trials. | Illustrations and model not available.  Discussed assumptions, confounding, lag times but not competing causes. | Method explained well.  Data sources explained |
| **Perreault, 1998[22]** | To compare average and marginal life-time cost-effectiveness of increasing dosages of statins for primary prevention of CHD | Canada, 1992?,  (not clear)  Hypothetical high risk (smoker, DBP>100mmHg) and low risk (non-smoker, DBP<80mmHg) men and women | Age, sex, cholesterol, HDL-C, diastolic blood pressure, left ventricular hypertrophy, glucose intolerance, smoking | No disease categories  No other treatments | Years of life saved, Cost per life years saved | Treatment with lovastatin at a dosage of 20mg/d apparently cost-effective in middle-aged men and women with baseline total cholesterol >6.67mmol/L. Treatments with 40mg/d is also cost-effective for total cholesterol >7.84mmol/L. However 80mg/d not cost-effective for primary prevention. | None  Validation was reported in previous papers | Illustrations& model not available.  Discussed assumptions, confounding, lag times & competing causes. | Narrow focus.  Not based on a real population.  Data sources adequate |
| **Grover 1999[23]** | To estimate cost-effectiveness of statin therapy in secondary prevention, using 4S study published results | Canada,  (Not clear)  M-F 40-70 yrs 15% random sample of LRC program | Mean BP, LDL/HDL, smoking, glucose intolerance | No disease categories  No other treatments | Recurrent CHD events  Cost per life year saved (LYS) | Suggested benefits of long-term statin therapy generally cost effective.  Costs for low risk patients with LDL/HDL>5 $5424 - $9548 per LYS in men and $8339-13747 in women.  In high-risk patients $4487 to 8532 in men and $5138 to 8389 in women. | One-Way sensitivity analysis  Model estimates were compared with events observed in 4S trial- predicted and observed rates were quite similar | Illustrations& model not available.  Discussed assumptions but not confounding, lag times or competing causes. | Limited to a very narrow focus  Little exploration beyond 4S trial  Data sources adequate |
| **Perreault, 1999[24]** | To estimate the potential effect of primary prevention treatment of hyperlipidemia or hypertension to reduce the risk of CHD death | Canada,  Years unclear 1986? 1992?,  M-F aged 35-74 (Canadian Heart Health Survey Population.) | Smoking, Cholesterol/HDL-C ratio, blood pressure | No disease categories  No other treatments | Number of CHD cases prevented | The clustering of modifiable risk factors in hypertensive patients demonstrated the need for comprehensive RF screening | No sensitivity analysis  Validation of the model checked previously | Illustrations& model not available.  Discussed assumptions, lag times, but not confounding or competing causes. | Adequate data quality for population, risk factors &treatment uptake. But data on treatment effectiveness relatively poor, ignored a meta-analysis. |
| **Lowensteyn 2000[25]** | To evaluate potential long term cost effectiveness of exercise training for cardiovascular disease risk factors | Canada, 1992, M-F aged 35-54, 55-64, 65-74 | Age, sex, HDL/LDL ratio, blood pressure, CVD presence, glucose intolerance, smoking | No disease categories  No other treatments | Years of life saved,  Cost per life years saved | Assuming 100% lifetime adherence, exercise training gained 0.7 life-years in men aged 35-54. Gains smaller in older men and women without CVD, but larger in those with CVD. Still cost effective. Assuming 50% adherence | One-Way sensitivity analysis  Validation of the model was checked previously (Grover 1998) | Illustrations& model not available.  Discussed assumptions & competing causes, but not confounding or lag times. | Clearly written paper.  Data adequate.  Sensitivity analyses well described |
| **Grover, 2001[26]** | To estimate long term cost effectiveness of lipid therapy among diabetic patients without CHD versus results in CVD patients without DM | Canada, USA, Italy, Spain, Germany France, 1998, M-F | Smoking, HDL-C, LDL-C, diastolic blood pressure, diabetes,  Diagnosed CVD at baseline | No disease categories  No other treatments | Years of life saved,  Cost per life years saved | Among diabetic men and women who do not have CVD, lipid therapy is likely to be as cost-effective as treating nondiabetic individuals with CVD | No sensitivity analysis  Calibration yes | Illustrations& model not available.  Discussed assumptions but not competing causes, confounding or lag times. | Assumed all subject were non-smokers!  Data sources presented but lacked RCT data on primary prevention in diabetes patients |
| **Grover 2003[27]** | To compare cost/effectiveness of lipid modification in primary prevention of CVD (with and without indirect costs) | Canada  Restricted to hypothetical cohort of 1000 participants reflecting baseline risk factor levels of the population | Smoking, LDL-C/HDL-C, mean blood pressure, glucose intolerance, age, sex | No specific disease categories,  10 mg daily Atorvastatin for CVD-free people at baseline. People categorised as: low risk: (Normal BP, non smoker) or -High risk: (High BP, smokers). | Direct and indirect cost per life year saved | Lipid therapy with statins can reduce CVD morbidity and mortality.  Adding indirect costs associated with productivity losses can result in cost savings to society. | One-Way sensitivity analysis | Illustrations& model not available.  Discussed assumptions, confounding & lag times but not competing causes | Data sources poorly explained |

Table 4. Summary table for CHD Policy Analysis Model

| **Author (year)** | **Purpose of the model** | **Model setting, Time period & Population** | | **Risk factors included** | **Disease categories & Treatments included** | **Outcomes studied** | **Key Results** | **Sensitivity analysis & Validity** | **Transparency** | **Strengths / Limitations &Data Quality** |
| --- | --- | --- | --- | --- | --- | --- | --- | --- | --- | --- |
| **Babad 2002[28]** | To evaluate the impact of different primary prevention strategies on health care costs | England and Wales, time not clear,  45-84 ? M-F | Smoking, cholesterol, systolic blood pressure, age, sex | | No disease categories  No other treatments | Stable angina, unstable angina, MI, Sudden death, stroke death, other CV death, cancer death, other death | None reported | No sensitivity analysis  No validation | Illustrations& model not available.  Discussed assumptions but not confounding, lag times or competing causes | Considers treatment adherence, compliance, treatment delay and effectiveness for risk factors. However limited number of risk factors.  Data quality adequate. National survey used to populate the model. However Framingham equations greatly over-estimate risk for English population |
| **Cooper 2002[29]** | To explore treatment, survival, and subsequent CHD event experience of CHD patients | England and Wales, time not clear, ...-85 M-F | No risk factors included | | Angina (stable/unstable), AMI, post MI,  CABG, & PTCA only, no specific medical therapies | Number of deaths prevented, morbidity prevented, CHD mortality, unstable angina admissions, patient investigations, angiograms, PTCA, CABG, noncardiac deaths | CABG: 19 deaths postponed/prevented per million population | No sensitivity analysis  Cardiac deaths estimated by the model were validated against data from ONS-model 12% underestimates. Angina prevalence estimate of the model compared with HSE 94 prevalence. Discrepancies ranged from 1% to 20% | Illustrations & model not available.  Discussed assumptions but not confounding, lag times or competing causes | Uncritical and rather arbitrary approach to selection of data sources. Data quality often poor. No attempt to quantify or manage uncertainties.  Future model versions to include secondary prevention? Model fit is better for men than women. |

Table 5. Summary table for IMPACT CHD Mortality Model

| **Author (year)** | **Purpose of the model** | **Model setting, Time period & Population** | **Risk factors included** | **Disease categories & Treatments included** | **Outcomes studied** | **Key Results** | **Sensitivity analysis & Validity** | **Transparency** | **Strengths / Limitations &Data Quality** |
| --- | --- | --- | --- | --- | --- | --- | --- | --- | --- |
| **Capewell 1999[30]** | To estimate proportion of CHD mortality fall in Scotland 1975-1994 attributable to treatments and to risk factor changes | Scotland, 1975-1994,  Population 5.1 million  M-F aged 45-64,65-74, >74 | Smoking, cholesterol, blood pressure, deprivation | Comprehensive  *See footnote | Deaths prevented or postponed (DPP) | 6205 fewer deaths observed in 1994 compared with 1975 base year.  Treatments together explained 40% of the mortality fall, 2722 DPPs (min 1373- max 5986);  Major Risk factors explained 51% of the fall: 4025 DPPs (3412-4679); 9% attributed to other, unmeasured factors | Multi way sensitivity analysis using - Analysis of extremes method  Validation: Estimated falls in CHD mortality were compared with observed falls in CHD mortality | Illustrations available & model available on request.  Discussed assumptions & confounding but not lag times or competing causes | Aims to include ALL effective CHD treatments given in 1994.  Mortality the only outcome. (Not non-fatal events)  Omits diabetes, BMI, Physical activity, diet, antioxidants, Barker.  Data quality adequate  (Census, MONICA, National population statistics, results from representative studies) |
| **Capewell 1999[31]** | To determine the extent to which increases in the uptake of effective treatments could further reduce CHD mortality in Scotland in 1994 | Scotland, 1994, all adults 45+ | No risk factors considered, apart from medications for hypertension | Comprehensive  *See footnote | Deaths prevented or postponed | 2722 DPPs between 1975 and 1994, attributable to treatments. (Min1373-max5986)  Increasing uptakes to 80% of eligible patients would have prevented or postponed approx 4078 DPPs (1886-6702). 39% from 2’prevention, 29% from Heart failure, 13% from initial treatments for AMI, 10% from HT and 8% from angina treatments. | Multi way sensitivity analysis using Analysis of extremes  Previously validated: comparison of estimated and observed falls in CHD mortality | Illustrations available & model available on request.  Discussed assumptions, confounding & lag times. Competing causes not relevant. | Considers a wide range of effective treatments available for CHD.  Outcome is mortality only (not nonfatal events).  Data quality adequate |
| **Capewell 2000[32]** | To determine how much of the recent CHD mortality fall in New Zealand can be attributed to treatments or to risk factor changes | Auckland 1982-1993,  Population 996,000 M-F | Smoking, cholesterol, blood pressure, statins for primary prevention, | Comprehensive *See footnote | Deaths prevented or postponed | 558 fewer deaths observed in 1993 than expected from 1982 rate. Medical and surgical treatments estimated to prevent or postponed 310 deaths (101-920), risk factor changes 361(204-596) DPPs  [Smoking 204, cholesterol 79 Diastolic BP 97 other factors 28] | Multi way sensitivity analysis- Analysis of extremes  Estimated falls in CHD mortality were compared with observed falls in CHD mortality | Illustrations available & model available on request.  Discussed assumptions, confounding, lag times& competing causes. | Considered a wide range of effective treatments for CHD. Outcome is mortality only. Nonfatal events not included. Omitted diabetes, BMI, Physical activity, diet, antioxidants, Barker  Data quality adequate |
| **Critchley 2003[33]** | To examine potential for risk factor changes to reduce CHD deaths in Scotland | Scotland, 1994-2010, M-F aged 45-84 | Smoking, cholesterol,  blood pressure | Treatments not  considered | Deaths prevented or postponed  (DPPs) | 2169 DPPs if recent risk factor trends simply continued to 2010. Additional, modest RF reductions would prevent 4749 deaths. [2167 cholesterol, 1168 smoking, 914 Diastolic BP reductions respectively]. Extrapolation to UK pop suggested approx. 53,000 lives could be saved in 2010. | Multi way sensitivity analysis- Analysis of extremes  Estimated falls in CHD mortality were compared with observed falls in CHD mortality | Illustrations available & model available on request.  Discussed assumptions, confounding, lag times& competing causes. | Considers major risk factors only, omits diabetes, BMI, Physical activity, diet, antioxidants, Barker.  Data quality adequate |
| **Critchley 2003[34]** | To estimate life years gained due to risk factor changes and improved treatments in Scotland between 1975-1994 | Scotland, 1975-1994,  M-F aged 45-84 | Smoking, cholesterol,  blood pressure | Comprehensive *See footnote | Life years gained (LYGs) | Treatments together prevented or postponed 1862 deaths. This resulted in 12025 LYGs (8689-14,461). Risk factor reductions prevented or postponed 2674 deaths resulting in 35991 LYGs (25782-40750). 50% from smoking.  70% of LYGs were in men. | Multi way sensitivity analysis- Analysis of extremes  Estimated falls in CHD mortality were compared with observed falls in CHD mortality | Illustrations available & model available on request.  Discussed assumptions, confounding, lag times& competing causes. | Considers a wide range of effective treatments for CHD. Mortality only. Nonfatal events not included. Omits diabetes, BMI, Physical activity, diet, antioxidants, & Barker. Data quality adequate |

***AMI:** Cardiopulmonary resuscitation, thrombolysis, aspirin, PTCA, Beta blockers, ACE inhibitors; **Secondary prevention (post MI/CABG/PTCA):** Aspirin, Beta blockers, ACE inhibitors, Statins, Warfarin, Rehabilitation; **Chronic angina:** CABG surgery, Angioplasty, Aspirin, Statins; **Unstable angina:** Aspirin, Aspirin & Heparin; **Heart failure:** ACE inhibitors; **Hypertension treatment**

Table 6. Summary table for The Global Burden of Disease Project

| **Author (year)** | **Purpose of the model** | **Model setting, Time period & Population** | **Risk factors included** | **Disease categories & Treatments included** | **Outcomes studied** | **Key Results** | **Sensitivity analysis & Validity** | | **Transparency** | **Strengths / Limitations &Data Quality** |
| --- | --- | --- | --- | --- | --- | --- | --- | --- | --- | --- |
| **Murray 1997[35]** | To provide a standardised approach to epidemiological assessments and use a standard unit, the DALY to aid comparisons. | World, 1990 | Smoking, blood pressure, physical activity | None  None | DALYs | Developed regions experience 12% of the burden and spend 90% of health care. CVD explain 10% of the total DALYs (20% in developed world, 23% in USSR & Eastern Block), 8% in developing countries. | | None  None | Illustrations & model not available.  Discussed assumptions & competing causes but not confounding or lag times. | Large and ambitious.  Data quality: Adequate for this model |
| **Murray 1997[36]** | To compare three scenarios of future mortality and disability for different causes and 8 regions of the world | World, 1990-2020 | Smoking | None  None | Deaths, DALYs, years of life lost | In established market economies life expectancy will be 88 for women and 78 for men. CVD will remain top cause of death globally. In 2020 CHD will account 20% of DALYs in EME counties and 15% in the world. | | Different scenarios (optimistic vs pessimistic) explored.  Calibration, Validity – not checked | Illustrations & model not available.  Discussed assumptions & competing causes but not confounding or lag times. | Useful for WHO and strategies. Includes trends for CHD and CVD however does not provide information on specific interventions.  Data sources adequate |
| **Ezzati 2002[37]** | To estimate contributions of selected risk factors to global and regional disease burden | World, 2000 | High blood pressure, high cholesterol, high BMI and smoking besides other 22 selected risk factors | None  None | DALY | Maternal and childhood underweight accounted 15% ; high blood pressure 4.4% (64 million), tobacco 4.1% (59 million), high cholesterol % 2.7 (40 million) of the global DALYs. | | None  None | Illustrations & model not available.  Discussed assumptions & competing causes but not confoundingor lag times. | Data sources adequate |
| **Murray 2003[38]** | To estimate health effects and costs of selected interventions to reduce the risks associated with high cholesterol and high blood pressure in different parts of the world | Three WHO regions (South east Asia, Latin America and Europe), 2000, Whole population | Cholesterol, SBO, smoking, BMI | None  Treatments? | Cost per DALY averted | All personal and non-personal interventions explored in this work were cost-effective over all three WHO regions.  In low resource settings population intervention strategies to lower salt intake, cholesterol concentration or both would be purchased first. Decision makers would next move to combined strategy of legislated reductions in salt content of processed foods with mass-media campaigns, and then add the absolute-risk approach to management of blood pressure and cholesterol concentration. | | Multi-way sensitivity analysis  Validity was not checked | Illustrations & model not available.  Discussed assumptions & competing causes but not confounding or lag times. | Useful primary prevention model. Focuses on effective interventions.  Detailed paper supported with web-tables to explain the method used.  Does not include effect of specific treatments.  Adherence omitted.  Limitations discussed briefly.  Data sources adequate |

Table 7. Summary table for other CHD policy models

| **Author (year)** | | **Purpose of the model** | | **Model setting, Time period & Population** | | **Risk factors included** | | **Disease categories & Treatments included** | | **Outcomes studied** | | | **Key Results** | | **Sensitivity analysis & Validity** | | **Transparency** | | **Strengths / Limitations &Data Quality** |
| --- | --- | --- | --- | --- | --- | --- | --- | --- | --- | --- | --- | --- | --- | --- | --- | --- | --- | --- | --- |
| **Kottke 1985[39]** | | To compare expected benefit of high-risk and population strategies on the basis of risk factor distributions of the male population of Eastern Finland | | North Karelia, Finland,  1992,  Men aged 25-59 in 1972 | | Cholesterol  Diastolic BP | | None  Targeting Cholesterol and DBP in NK men. | | Number of deaths prevented | | Three strategies explored.  **-Achieved** (reducing cholesterol 10% and reducing DBP below 95mmHg)  **-Good** (reducing cholesterol 20% and reducing DBP below 90 mmHg)  **-Ideal** (reducing cholesterol below 190, and reducing DBP below 80).  Implementing these 3 (achieved/ good/ ideal). Interventions in **a) High-risk** people would reduce population CVD by 16%, 28% and 33% respectively  b) **Population level** would reduce population CVD by 31%, 52% and 70%. | | | None  Calibration: used actual mortality rates for NK cohort | | Illustrations & model not available.  Discussed assumptions & competing causes but not confounding or lag times. | | Did not acknowledge any limitations  Data quality good  However study limited to men aged 25-59 years |
| **Nissinen 1986[40]** | | To estimate costs and effects of North Karelia Project hypertension control program during the first 5 yrs | | Finland, North Karelia,  1972-1977  M-F 35-64 yrs | | Hypertension | | None,  No other treatments | | Life years gained life expectancy, & costs | | 1239 MI deaths and 327 stroke deaths observed between 1972 and1977. 288 fewer than expected: 134 were attributable to BP treatment. Thus 2143 life years saved; cost per QALY was $3612 (excluding earnings) and $322 (including earnings). | | | None  None | | Illustrations & model not available.  Discussed assumptions & competing causes but not confounding or lag times. | | Rather old paper  Data sources adequate |
| **Browner 1986[41]** | | To estimate the overall impact of a risk factor modification programme | | USA, 1964-1974 and 1972-1983, men only aged 35-59 | | Cholesterol | | None,  No specific treatments | | Incidence | | With optimistic assumptions about the impact of cholestyramine Rx at various cholesterol. levels about 5% of CHD cases in middle-aged men could be prevented. More realistic assumptions reduced that estimate by half. | | | None  None | | Illustrations & model not available.  Discussed assumptions but not competing causes, confounding or lag times. | | Simplistic model. Model details not clear.  Focused only on cholesterol.  Model mostly used data from a small trial therefore issues of generalisability  poor |
| **Hjort 1986[42]** | | To examine potential benefits of secondary prevention after AMI | | Norway, 1980?  (Not clear),  MI survivors aged 20-75 yrs | | None | | Post MI  Bet-blocker | | Life years gained | | Beta-blockers gained 0.25-1.6yrs in post MI patients.  But Smoking cessation has similar effect and lasts longer:  Smoking prevalence 50%, If all quit, 5120 extra survivors and 3.3 LYG gained per quitter. | | | None  None | | Illustrations & model not available.  Discussed assumptions but not competing causes, confounding or lag times. | | Very simple modelling methodology  Data: Main data source was Timolol RCT hence generalisabilitypoor |
| **Kottke 1988[43]** | | To examine the magnitude of risk factor clustering within individuals and to examine the implications of risk factor clustering for CHD prevention policy | | US railroad (USRR) cohort and Finnish North Karelia cohort  USRR-1959-‘64,  NK: 1972-78  US railroad cohort- 2571 men aged 40-59 (1959).  North Karelia 3022 men aged 40-59 | | Smoking, cholesterol, blood pressure | | No specific treatments  No groups | | Number of deaths, number of morbidity, non fatal AMIs | | Reducing cholesterol 10%, smok 20% and DBP 5% in general population would lower CHD death by 33%-38% & nonfatal AMIs by 21% -23% (US-NK)  Reducing cholesterol 4%, smok 15% and DBP 3% in general population would lower deaths by 22-18%. & nonfatal AMIs by 12-13%  Single interventions in high-risk groups were less effective i.e. max of 8% reduction in MI or CHD death. | | | None  None | | Illustrations & model not available.  Not discussed assumptions, competing causes, and confounding or lag times. | | Limitations in data sources.  US railroad cohort:  Selected- healthy worker effect?  Generalisibility to US population is questionable.  North Karelia results more generalisable. |
| **Park 1989[44]** | | To compare high risk versus population approach to primary prevention in North Karelia | | North Karelia, Finland, 1972-77 | | Cholesterol: simulated reduction to 180mg/dl a) in high risk b) in whole pop.  Blood pressure | | None, None | | Number of deaths prevented | | Reducing cholesterol below 180 in top quartile would lead to 34% reduction in CHD deaths. Reducing cholesterol 30mg/dl across pop would lead 24% reduction in CHD deaths. | | | None  None | | Illustrations & model not available.  Discussed assumptions & competing causes but not confounding or lag times. | | Poor-early model. Lacks detail.  Data quality is adequate. North Karelia data were used. |
| **Martens 1990[45]** | | To assess cost effectiveness of cholesterol reduction with simvastatin or cholesteramine | | Holland, 1988, Dutch pop aged 35-70 | | Cholesterol | | None, None | | Number of CHD events prevented, life expectancy, cost per life years gained | | Simvastatin increased the life expectancy in men and women more than 3 times those with cholestyramine. Simvastatin therapy for men cholesterol>8 mmol/dl increased life expectancy by 1.75 yrs whereas cholestyramine increased 0.48 yr.  For men with initial cholesterol >8 mmol/l the cost of life years saved of cholestyramine ranged $104,000 to $241,500. For simvastatin cost effectiveness ranged by $23,000 to $49,000. | | | One-way sensitivity analysis  None | | Illustrations & model not available.  Discussed assumptions but not competing causes, confounding or lag times. | | Poor- very narrow. Only simvastatin or cholestyramine. Focused mainly on extremely high chol levels 7,8,9.  Data sources poor |
| **Zhuo 1991**  **CRISPERS** | | To investigate the relationship between the risk coefficients and simulation outcomes using CRISPERS model | | Model was developed in Canada using North Karelia population, 1972-1979, 3022 men aged 40-59 | | Cholesterol, number of cigarettes, DBP | | None, None | | CHD mortality, MI incidence, deaths due to other causes | | All population approaches are better than high-risk approaches. | | | Multi-way- discussed  Validity -checked | | Illustrations & model not available.  Discussed assumptions & competing causes, but not confounding or lag times. | | Early modelling study. Includes only young men  Data: Adequate |
| **Johannesson 1991[46]** | | To estimate cost-effectiveness of CVD prevention | | Sweden, 35-74 yr M-F free of CVD | | Smoking, cholesterol, blood pressure, glucose intolerance, LVH | | Angina, AMI, sudden death,  None | | Life years gained, cost | | In this paper they described the model only no results were reported | | | None  None- but planned | | Illustrations available but model not available.  Discussed assumptions but not competing causes, confounding or lag times. | | Data: Adequate |
| **Macksikak 1992** | | To predict disease rates in cohorts and to estimate effect of interventions | | New South Wales? Australia, Hypothetic cohort of 100,000 men aged 40-64 | | None | | AMI, sudden death, CHD death, death from other causes (7 CHD states were described)  No treatment | | CHD incidence, AMI | |  | | | Sensitivity None  Validity None | | Illustrations & model not available.  Not discussed assumptions, competing causes, lag times or confounding. | | Relatively narrow age group. Only men included. Arbitrary chosen changes in management and natural history of CHD.  Data sources adequate; national surveys and MONICA were used. |
| **Jones 1994** | | To quantify cost-benefit relationship of walking to prevent CHD | | USA, time? hypothetical cohort of M-F aged 35-74. | | Physical activity (Assumed RR=1.9, 95%CI 1.5 to 2.4) | | None | | Net cost | | At a RR of 1.9 for heart disease associated with sedentary behaviour, $5.6 billion would be saved annually if 10% of adults began regular walking program.   If all currently sedentary people (40%) start to walk 5-hours/per week then this would save annually $4.3 billion, mostly in men.  Walking is beneficial for men aged 35 to 64 yrs and for women aged 55 to 64 yrs. | | | One-way SA,  None | | Illustrations & model not available.  Not discussed assumptions, competing causes, confounding or lag times | | A poor study. No details of methods on calculating the reductions of events or deaths.  Data sources described poorly |
| **Doliszny 1994[47]** | | To assess the possible contribution of CABG o the decline in CHD mortality between 1970 and 1984 in Minneapolis | | Minneapolis St Paul, USA, 1970-1984, 30-74 M-F | | Smoking, BP, DM | | Angina, AMI, post MI, heart failure, CABG  CABG surgery | | Number of deaths prevented | | CABG contributed modestly to the decline in CHD mortality 1970-84. By 1984, the estimated contribution had increased to 6.6%. Adjustment 'attenuate but does not eliminate' this contribution. It is questionable whether contribution increased 1980s and 1990s because of competition from other therapeutic approaches. | | | None  Split 2/3 and compared predicted number of deaths with observed deaths- there was a good agreement | | Illustrations available but model not available.  Discussed assumptions but not competing causes, confounding or lag times | | Clear well written paper. However very narrow, only looked a CABG. Used entirely different method. Useful method for validation.  Data quality adequate |
| **Silagy 1994[48]** | | Modelling workload from different servicing options in primary care, in order to avert future CHD events. | | South of England, 1992,  5,727 M-F, aged 35-64 registered with GPs | | Smoking, cholesterol, HDL, blood pressure, diabetes, LVH | | None, None | | CHD events | | 517 of the population predicted to get CV event within 10 years (242 in men, 189 in women).  a) No screening: 5035 patients need intervention to avert 73/517 CVD events b) limited screening would avert 29/73 events  c) extended screening would avert 48/73 events | | | None  None | | Illustrations & model not available.  Discussed assumptions but not competing causes, confounding or lag times | | This model looks at CHD events not deaths. Includes GP registered patients not the real population. Theoretical interventions not real.  Data quality adequate |
| **Bonneux 1994[49]** | | To make quantitative analysis of the dynamics of the heart disease epidemic to explore future of heart disease morbidity in the Netherlands | | Netherlands, 1985-2010, Dutch population | | None | | Angina, AMI, post MI, HF  None | | CHD incidence, prevalence, mortality | | Prevalence rates of CHD will decrease among the young and middle aged but increase among elderly by 2010 | | | Extreme scenarios  Model estimates on hospital admission and hospital deaths due to CHD were compared with observed figures in 1985. Looks suspiciously good. | | Illustrations & model not available.  Discussed assumptions & competing causes, nut not confounding or lag times | | Omits sudden deaths. Only looked at hypothetical changes in incidence and survival rates to 2010. Did not consider RFs or Rx.  Data: National statistics were used, adequate |
| **Bensley 1995[50]** | To quantify the potential health gain in terms of a fall in mortality from prevention and treatment policies | | Yorkshire- UK, 1989, M-F 35-74 | | None | | Angina, AMI, sudden death, CABG, PTCA, post MI  None | | Number of CHD deaths, number of CABG, PTCA and angiograms, and costs associated with them /GP referrals | | 1-Model can be used to examine sensitivity of outputs (deaths) to parameters: attack rate, referral rate, and incidence. 2-Forecasting effects on future deaths of hypothetical reductions in attack rates, SVD rate, hospital death rate and episode rate e.g.. | | | One way sensitivity analysis - discussed reasonably but without examples  Numbers predicted by model was compared observed figures in Yorkshire but no further detail provided | | Illustrations available but model not available.  Discussed assumptions but not competing causes, confounding or lag times | | Simple tool to communicate with decision makers, model was built with consulting practising clinicians. However does not include risk factors, data sources rather old and needs to be updated. Model focuses on revascularisation only; other CHD treatments overlooked i.e. statins. Model does not include heart failure. | |
| **Pharoah 1996[51]** | | To estimate cost-effectiveness of statins in lowering serum cholesterol in people with varying CVD death risk | | UK, Cambridge and Huntington, 10 years period, M-F 45-64 | | Cholesterol (fifths), | | None  Statins for primary prevention | | Number of CHD deaths prevented, number of CHD cases prevented, and costs per life year saved | | The average cost effectiveness for statin therapy for 10 years, in men aged 45-64 with no history of CHD but cholesterol>6.5 mmol/L was £136,000 per life years saved. It was £32,000 with pre-existing CHD and cholesterol>5.4 mmol/L. Cost effectiveness change widely with existing CHD history and cholesterol levels. In women aged 45-54 with angina and cholesterol 5.5-6.0 mmol/l CE: £361,000 | | | Multi-way - Different scenarios explored  None | | Illustrations available but model not available.  Not discussed assumptions, competing causes, confounding or lag times | | It was assumed a typical treatment cost for statins for patients. So the compliance would be the same as reported in studies  Data quality adequate |
| **Oster 1996[52]** | | To estimate the effects of reducing dietary saturated fat intake on the incidence and economic cost of CHD | | USA, 1990-2000,  35-69 yrs persons with cholesterol level of 5.17 mmol/l or higher, free of CHD | | Cholesterol, saturated fat intake (Strategy: reducing saturated fat intake by 1 or 3% | | None, None | | Incidence (CHD) lifetime direct and indirect costs | | Reducing dietary saturated fat intake by 1 or 3% in people with high cholesterol levels would reduce CHD incidence by 32,000 and 199,700 respectively and would save $4.1 and $12.7 billion over 10 years. | | | None  None | | Illustrations& model not available.  Discussed assumptions but not competing causes, confounding or lag times | | Estimates direct and indirect costs. However it just evaluated impact of diet-saturated fat change through cholesterol, other factors ignored.  Data sources adequate |
| **Bots, 1996[53]** | | To study relative contributions of medical care and changes in CVD risk factors to decline in CHD mortality in Netherlands between 1978 and 1985 | | Netherlands, 1978-1985,  Dutch pop- age-sex not clear | | Smoking, cholesterol, blood pressure | | AMI, Post MI  Beta blockers, anticoagulants, ant platelets, CABG, CCU care | | Number of deaths prevented | | Treatments accounted 46% and risk factor changes 44% of the CHD mortality decline in Netherlands between 1978-85 | | | None  Estimated figures compared with observed deaths prevented- 90% explained | | Illustrations available but model not available.  Discussed assumptions, confounding, lag times but not competing causes. | | Estimates are crude, not age-sex specific- overlaps between treatments and risk factors not accounted/ mean blood pressure change not considered- PAR method was used  Overestimation??  Data sources adequate |
| **Lightwood, 1997[54]** | | To estimate short term benefit of smoking cessation | | USA, 1990, 35-64 yrs M-F | | Smoking  1% absolute reduction in smoking prevalence | | None, None | | Number of deaths prevented, hospital admission for CHD and CVD | | A national programme that would reduce smoking prevalence by 1% per year would prevent 98 100 hospitalisations for AMIs and strokes (and 1300 deaths of AMI outside the hospital) and eliminate the need to spend ~$3.2 billion on the treatment of MI and strokes for 7 years. | | | None  None | | Illustrations& model available.  Discussed assumptions, lag times but not confounding or competing causes. | | Very narrow- smoking cessation in prim prevention. -No sensitivity analysis  Data sources adequate |
| **Kellet 1997[55]** | | To estimate likely gains in life expectancy of patients with CAD treated with statins | | CHD patients who had 2, 3 vessel and left main stem coronary artery disease, aged 40-80 | | None | | CHD patients who had 2, 3 vessel and left main stem coronary artery disease  Two treatment options were possible: medically or surgically | | Quality adjusted life expectancy | | Statins were estimated to provide a gain in life expectancy for medically managed patients of all ages with CHD, ranging from 4.6 to 10.1 QALY | | | None  None | | Illustrations& model available.  Discussed assumptions but not lag times, confounding or competing causes. | | Very narrow purpose, decision tree analysis.  Data sources adequate |
| **Riviere 1997[56]** | | To determine the cost effectiveness of simvastatin in the secondary prevention of CHD in Canada | | Canada, …. Average 59.4 years old M-F | |  | | None, None | | Cost per life year saved | | Premise A: no benefit beyond 5.4 yrs and survival curves continue parallel for 15 yrs.  Premise B: The benefit from statins is cumulative, survival curves diverge to 10 yrs then continue parallel until 15 yrs.  Premise C: The benefits of statins are assumed to continue for 15 yrs, survival curve would diverge for 15 yrs.  For Premises B and C cost effectiveness ratios estimated were $9,867 and $6,108 respectively. | | | One-way sensitivity analysis  None | | Illustrations& model available.  Discussed assumptions, but not lag times, confounding or competing causes. | | Clear plausible consistent with other studies however includes just one disease and one treatment  Data: Adequate  However treatment effectiveness was based only on 4S study. |
| **Bonneux, 1998[57]** | | To examine whether elimination of fatal diseases will increase health care costs | | Netherlands, 1986-1990, Dutch population | | None | | None  None | | Life expectancy and life time health care cost | | Elimination of CHD would increase life expectancy by 1.9 yrs proportion of CHD in lifetime health costs increase from 2.5% to 6%. The medical cost of added life years would be about £890 to £1400 per life year. | | | None None | | Illustrations available but not model.  Discussed assumptions& confounding but not lag times or competing causes. | | Data Sources: Based on old data for life expectancy. Poor.  Only medical costs estimated. |
| **Galgali 1998[58]** | | To calculate the potential for prevention by limiting the pop exposure to common risk factors | | New Zealand, 1992-97,  M-F 55+ yrs | | Smoking, cholesterol, hypertension, physical activity, obesity | | None, None | | Number of CHD deaths reduced | | 3% reduction in smoking, 3% reduction in hypertension, 6% decrease in physical inactivity, and 3%reduction in high cholesterol (in next 5yrs) would result in 1,228 fewer deaths per year (in next 5 yrs). | | | None  None | | Illustrations available but not model.  Not discussed assumptions, confounding, lag times or competing causes. | | Population and mortality data is national- adequate. However RRs based international studies. Could be a limitation. |
| **Augustovski 1998[59]** | | To evaluate the effects of aspirin in primary prevention of CVD patients with different risk profiles | | ? 10 years period (cohorts follow up),  - 7 hypothetical cohorts, M-F, age 55-65 with different CVD risk levels | | None (but cholesterol, HDL, SBP, smoking, DM, LVH were used to divide the subjects in risk categories) | | None  Aspirin 75-375 mg | | QALY (end point: CHD or stroke event | | Lowest risk cohort (just high cholesterol) would have a loss of 1.8 days QALY. High risk cohort (all rfs +) would achieve 11.3 days QALY | | | One-Way sensitivity analysis  Cox regression analyses for validity | | Illustrations & the model not available.  Discussed assumptions but not confounding, lag times or competing causes. | | Aspirin effectiveness is assumed to be same in women. Compliance ignored, generalisability? For which pop?  Data: Adequate |
| **Bonneux 1999[60]**  (Incidence-prevalence-mortality model) | To estimate changing prevalence of CHD as a consequence of the changing disease history. | | Life table population is used for estimations. 10 yrs period-  Dutch M-F aged 40-80 in 1980-83 / 1990-93. | | None | | ACE, recurrent MI, CHD death, CVD deaths, deaths other than CHD | | Incidence, prevalence, CHD deaths, hospital discharges | | Incidence of first ACE changed little between 1980-83 and 1990-93 among men<60 incidences decreased by 10% among women incidence increased by 9%. Average prevalence of survivors increased sharply, predominantly among elderly: from 12% in 1980s to 16% in 1990s in men >60 and 3.3% to 5.5% in women >60. | | | One-way sensitivity analysis- using plausible recurrence probabilities | | Illustrations & the model not available.  Discussed assumptions but not confounding, lag times or competing causes. | | Quite broad CHD definition- no detail. Details available from a web page.  Data sources reported. Administrative data were used. | |
| **Mui 1999[61]** | To project the incidence rates for CHD, and the number of CHD cases, hospital costs for M/F aged 45-69 up to 2014 | | Australia, 1989-2014, M-F aged 45-69 | | Smoking, cholesterol, TC/HDL ratio, blood pressure | | None, None | | Incidence (CHD and stroke) | | If current CHD risk factor distributions change only by a location shift and the treatment availability stays the same CHD incidence will decline 13% in M and 24% in W by 2014 | | | None  Model CHD incidence estimates were compared with MONICA data for the same year. Model 50% overestimates in men and 110% underestimates for women | | Illustrations & the model not available.  Discussed assumptions but not confounding, lag times or competing causes. | | Model takes into account changes in risk factors with aging, change in smoking status. Model validity was improved with regressing the rates. Estimates hospitalisation costs.  Data: Adequate | |
| **Thompson 1999[62]** | | To explore lifetime health and economic consequences of obesity using a lifetime model. | | USA, ?, M-F aged 35-64 | | Smoking, cholesterol, DBP, diabetes, obesity, age, sex | | None  None | | Number of CHD, stroke event, life expectancy | | Lifetime risk of HT, high cholesterolemia, DM, stroke and CHD in men 45-54 increases with BMI.  Life expectancy reduced by 1 year in men and women with BMI over 37.5.  Lifetime medical care costs for treatment of (High cholesterol, HT, DM, CHD, stroke) are estimated to differ by $10,000 ($29,600 vs $19,600). | | | None  None | | Illustrations & the model not available.  Discussed assumptions but not confounding, lag times or competing causes. | | Data sources reported poorly |
| **Lindholm 1999[63]** | | To estimate C/E calculation within a defined budget for CHD primary prevention options BP, Cholesterol, treatments and community interventions | | Vaterbotten, Sweden, 1996?, M-F 30-69 | | Smoking, cholesterol, BP, community based interventions (education and screening) | | None, None | | Cost per life year saved | | Hypertension treatment cost depends on cost of drug used. Marginal C/E ratios 17,000-500,000 ECU/life years saved (mean 55,000 Ecu).  Cholesterol lowering drugs 53,000-800,000 Ecu/LYS (mean 83,000 Ecu).  Community programme 4,000-23,000 Ecu/ LYS (mean 12,000 Ecu). Optimal division of resources would require lowering budget for hypertension treatment but increasing cholesterol treatment and community interventions. | | | One-way sensitivity analysis  Based on actual budget of 6.2million ecu | | Illustrations & the model not available.  Discussed assumptions but not confounding, lag times or competing causes. | | Basic model. Assumed no benefits from statins  Data: Adequate |
| **Naidoo 2000[64]** | | To simulate effects of achieving two targets of reducing smoking in terms of AMI and stroke hospitalisation numbers prevented | | England, 1996-2010, M-F aged 35-64 | | Smoking  Target1: reducing smoking prevalence from 28%(1996) to 26% (2005) and to 24%(2010)  Target2: More ambitious reductions- from 28%(1996) to 22%(2005) and to 17% (2010) | | None | | Number of AMI and stroke hospitalisations prevented | | Target1 would result in 347 AMIs and 214-stroke hospitalisation prevented in 2000 and 6386 AMIs; 4964 strokes in year 2010.  Achieving target 2 would result in 739 AMI and 11,304 strokes in 2010.  Target 1 would save £524 million and Target 2 would save 1.14 billion NHS costs. | | | One way sensitivity analysis  None | | Illustrations & the model not available.  Not discussed assumptions, confounding, lag times or competing causes. | | Data: Adequate |
| **Kuulasma 2000[65]** | | To explain the extent to which risk factor changes explain the variation in CHD events rate trends across 38 populations | | 38 populations over 30 countries, mid 1980s- mid 1990s, M-F aged 35-64 | | Smoking, total cholesterol, SBP, BMI) | | In another paper they considered immediate treatments for AMI and secondary treatment for post AMI patients. | | Change in CHD event rates | | During the study period risk scores and CHD event rates decreased. Trend model showed a poor fit, but after considering 4 years lag improved. The explanatory power of the analyses was limited (46% in M and 19% in W) by imprecision of the estimates and homogeneity of trends in the study populations. | | | One-way sensitivity analysis  Validity was checked by comparing estimated falls with observed falls in event and deaths. | | Illustrations available but the model not.  Discussed assumptions, confounding, lag times but not competing causes. | | Weak study design (ecological)  Data: From real populations and quality checked therefore adequate. |
| **Fichtenberg 2000[66]** | | To estimate whether California Tobacco Control Programme was associated with lower rates of CHD deaths | | USA, California, 1977-1997,  US California, CHD death rates | | Smoking | | None,  None | | CHD mortality | | Between 1989 and 1992 per capita cigarette consumption declined faster in California by 2.72 pack per year. IHD mortality also declined faster by 2.93/10000 per year. Programme was associated with 33,000 fewer CHD deaths 1989-1997. Effectiveness diminished after 1992 associated with 8,300 more deaths. A large aggressive tobacco control programme was associated with a substantial reduction in deaths from heart disease. | | | None  None | | Illustrations & the model not available.  Discussed assumptions & method refined for confounding and lag times. Not discussed competing causes. | | Quite simple model looked at all heart disease mortality.  Data: Adequate |
| **McNeil 2000[67]** | | To model life time risk of fatal or nonfatal CHD in different risk percentiles | | Australia, 1993-1995, Men aged 20-69 Australia | | Cholesterol | | None, None | | Number of CHD deaths prevented | | Top decile of individuals contained 23% of CHD risk, top 30% contained over 50%. Survival curves separate after age 45 in men, 55 in women | | | None  Predicted individual risk event compared with observed rate in AFCAPS and TEXCAPS primary prevention trials | | Illustrations available but the model not.  Discussed assumptions but not confounding, lag times or competing causes. | | Poor- lots of assumptions- risk at 69 extrapolated to 84  Data: Adequate |
| **Baker 2000[68]** | | To estimate the impact of using thresholds based on absolute risk of CVD to target drug treatment to lower blood pressure in the community | | Auckland/ New Zealand, ??, 2158 M-F aged 35-79 sampled from general population | | Smoking, cholesterol/HDL, SBP, diabetes, age and sex | | No disease categories included.  Antihypertensive treatments | | Number of CVD prevented (angina, MI, CHD death, stroke, TIA, congestive HF, peripheral vascular disease) | | 46,374 (12%) Auckland residents aged 35-79 receive antihypertensive treatment. It was estimated that 1689 disease over 5 yrs would be averted. Restricting treatments to individuals with >170/100mm/hg and BP between 150/90-169/99 mmHg who have 5 yrs disease risk >10% would avert 19,401. Implementing guidelines and use treatment thresholds based on absolute risk could significantly improve efficiency of antihypertensive treatments. | | | None  None | | Illustrations & model not available.  Discussed assumptions but not confounding, lag times or competing causes. | | Risk factor information was available from a national population study. Excluded Maori& Pacific Islander population. Confined to BP only. Assumed 25% of relative risk reduction, might be a high efficacy.  Data sources adequate |
| **Selmer 2000[69]** | | To estimate health and social consequence of reducing daily salt intake by 6 gr per person | | Norway, 1995-2020, over 40 M-F | | Blood pressure | | None, None | | Cost | | A 2 mmHg reduction in SBP would reduce stroke by 4.2%, MI by 3.8%. Implies overall 1-2% reduction in total mortality, Life expectancy in 40-year-old men increase 1.8 months, in women 1.4 months.  *25 year benefit:* 7000 lower MI deaths, 4500 lower stroke deaths, 87000 LYG (6000 lives saved at year 25) on average 10 LYGs each. Total 150000 LYG (discounted 52000). Net saving $270 million- 120 million discounted) | | | One-way sensitivity analysis  None | | Illustrations & model not available.  Discussed assumptions & competing causes but not confounding or lag times. | | Narrow  Data quality adequate with limitations on generalisability of FINMARK Study to Norwegian pop. |
| **Malik 2001[70]** | | To assess cost effectiveness of ramipril in patients with low, medium, high risk | | UK, 1998, men average age 66 from HOPE Trial | | None | | Highest risk, high risk and low risk patients.  Treated with ramipril | | LYG, cost per LYG | | Cost effectiveness of ramipril was £36600 £13600 and £4000 per life year gained at five years and £5300, £1900, and £100 per life year gained at 20 yrs in low, medium and high risk groups respectively. Treatment of medium risk HOPE population would cost UK NHS £360 million but would prevent 12 000 deaths. | | | One-way sensitivity analysis  None | | Illustrations & model not available.  Discussed assumptions but not competing causes, confounding or lag times. | | Data: Adequate |
| **Peeters 2002[71]** | | To measure CVD burden of disease in Framingham cohort, by generating years of life lost or lived with the disease | | USA, 1941-1991, M-F, aged 18-62 years at onset | | None | | Angina, AMI, heart failure, stroke, TIA, intermittent claudication | | Life expectancy and lives lived with disability | | 5,070 individuals without CVD at the beginning, 50% developed CVD and 60% died over 40 years.  34% developed CHD, 20% AMI, 14% stroke, and 14% heart failure.  Using life tables it is estimated that at age 50 20% (6.3 yrs for men and 5.7 yrs for women) of a populations residual life expectancy is spent with CVD. Much of this (4.7 yrs male, 3.7 yrs females) spent on CHD. | | | None  None | | Illustrations available but model not.  Discussed assumptions & competing causes but not confounding or lag times. | | Disease states are not mutually exclusive.  Data: Quite few event numbers for women. |
| **Marshall 2002[72]** | | To develop a model to evaluate costs and health benefits of implementing guidelines for prevention of CVD prevention--6 strategies (2 from joint British recommendations) | | England, 1998, Hypothetical cohort of 2000 patients aged 30-74 | | Smoking, cholesterol, HDL, blood pressure, diabetes, age, sex, LVH. | | None,  Aspirin, thiazid, Beta blocker, ACE inhibitor, statin | | Cardiovascular event prevented | | Novel strategies prevent more CVD at lower cost than traditional strategies | | | One-way sensitivity analysis  None | | Illustrations & model not available.  Discussed assumptions & competing causes but not confounding or lag times. | | Lacks detail on model, not very relevant on explaining risk factor or treatment effects on CVD in the population  Data sources adequate |

Appendix 4. List of excluded studies (Alphabetical order)

| **EXCLUDED STUDIES** | **Reason for Exclusion** |
| --- | --- |
| **Assman 1990[73]** | This is an individual data based statistical model. It is simply a validation study of PROCAM equations using Helsinki Heart Study. |
| **Avins 1998[74]** | This is a general evaluation of interventions used in North Karelia and Minnesota Heart Health Programmes. No CHD outcomes reported. |
| **Beard 1989[75]** | Not a modelling study. It is a case control study, which uses PAR estimations. |
| **Bronnum-Hansen 1999[76]** | No CHD outcomes reported |
| **Cleland 1998[77]** | This is an individual based statistical model not a population based CHD health policy model. |
| **Cowen 1996[78]** | CHD is not an outcome in this model, it is an input |
| **Glick 1992[79]** | Not a population based model. |
| **Grover 1994[80]** | Review, not an original model paper. |
| **Gunning-Schepers 1999[81]** | General editorial, not an original paper |
| **Gunning-Schepers 1987[82]** | General review, not an original paper |
| **Hatziandreu 1988[83]** | Decision analysis of hypothetical cohort of 1000 men aged 35 years followed up for 30 years. Not population based model. |
| **Hinzpeter 2000[84]** | German language. Abstract checked. Cost estimates only. |
| **Kaplan 1988[85]** | This study used logistic regression analysis and compared two cohort studies. It is not a population CHD health policy model. |
| **Kawachi 1990[86]** | Outcome is not CHD |
| **Kaplan 2001[87]** | No CHD outcome reported |
| **Liew 2002[88]** | This is a general review paper not an original paper |
| **Murray 1994[89]** | This not a CHD health policy model. This paper aims to explore ways of comparison between intervention and control communities in community intervention trials |
| **Murray 1994[90]** | This paper presents the distribution of DALYs by cause, age, sex and region. However provides very little detail on CVD. |
| **Murray 1994[91]** | This paper simply defines the cause of mortality by eight regions of the world, it does not model CHD. |
| **Nissinen 1992[92]** | Duplicate paper of Nissinen 1986[40] |
| **Peeters 2003[93]** | This is not a population CHD health policy model. It is an individual based statistical model |
| **Petersen 1982[94]** | Just reports on RF distributions and individual risk of developing CHD risk. This is not a population CHD health policy model. |
| **Salonen 1986[95]** | General evaluation of community based CHD control programmes |
| **Wolfson 1994[96]** | The Population Health Model (POHEM) was developed for chronic diseases but then never used for CHD policy analyses. This paper provides a framework to understand the POHEM Model. |

References

1. Goldman L, Cook E: **The decline in ischemic heart disease mortality rates. An analysis of the comparative effects of medical interventions and changes in lifestyle.** *Ann Intern Med* 1984, **101:** 825-836.

2. Weinstein MC, Coxson PG, Williams LW, Pass TM, Stason WB, Goldman L: **Forecasting coronary heart disease incidence, mortality, and cost: the Coronary Heart Disease Policy Model.** *Am J Public Health* 1987, **77:** 1417-1426.

3. Goldman L, Weinstein MC, Williams LW: **Relative impact of targeted versus populationwide cholesterol interventions on the incidence of coronary heart disease. Projections of the Coronary Heart Disease Policy Model.** *Circulation* 1989, **80:** 254-260.

4. Tsevat J, Weinstein MC, Williams LW, Tosteson AN, Goldman L: **Expected gains in life expectancy from various coronary heart disease risk factor modifications.** *Circulation* 1991, **83:** 1194-1201.

5. Goldman L, Weinstein MC, Goldman PA, Williams LW: **Cost-effectiveness of HMG-CoA reductase inhibition for primary and secondary prevention of coronary heart disease.** *JAMA* 1991, **265:** 1145-1151.

6. Hunink MG, Goldman L, Tosteson AN, Mittleman MA, Goldman PA, Williams LW *et al*.: **The recent decline in mortality from coronary heart disease, 1980-1990. The effect of secular trends in risk factors and treatment.** *JAMA* 1997, **277:** 535-542.

7. Tosteson AN, Weinstein MC, Hunink MG, Mittleman MA, Williams LW, Goldman PA *et al*.: **Cost-effectiveness of populationwide educational approaches to reduce serum cholesterol levels.** *Circulation* 1997, **95:** 24-30.

8. Goldman L, Coxson P, Hunink MG, Goldman PA, Tosteson AN, Mittleman M *et al*.: **The relative influence of secondary versus primary prevention using the National Cholesterol Education Program Adult Treatment Panel II guidelines.** *Journal of the American College of Cardiology* 1999, **34:** 768-776.

9. Phillips KA, Shlipak MG, Coxson P, Heidenreich PA, Hunink MG, Goldman PA *et al*.: **Health and economic benefits of increased beta-blocker use following myocardial infarction.[comment].** *JAMA* 2000, **284:** 2748-2754.

10. Prosser LA, Stinnett AA, Goldman PA, Williams LW, Hunink MG, Goldman L *et al*.: **Cost-effectiveness of cholesterol-lowering therapies according to selected patient characteristics.** *Ann Intern Med* 2000, **132:** 769-779.

11. Goldman L, Phillips KA, Coxson P, Goldman PA, Williams L, Hunink MG *et al*.: **The effect of risk factor reductions between 1981 and 1990 on coronary heart disease incidence, prevalence, mortality and cost.** *J Am Coll Cardiol* 2001, **38:** 1012-1017.

12. Tice JA, Ross E, Coxson PG, Rosenberg I, Weinstein MC, Hunink MG *et al*.: **Cost-effectiveness of vitamin therapy to lower plasma homocysteine levels for the prevention of coronary heart disease: effect of grain fortification and beyond.** *JAMA* 2001, **286:** 936-943.

13. Gaspoz JM, Coxson PG, Goldman PA, Williams LW, Kuntz KM, Hunink MG *et al*.: **Cost effectiveness of aspirin, clopidogrel, or both for secondary prevention of coronary heart disease.[comment].** *New England Journal of Medicine* 2002, **346:** 1800-1806.

14. Buck D, Godfrey C, Killoran A, Tolley K: **Reducing the burden of coronary heart disease: health promotion, its effectiveness and cost.** *Health Education Research* 1996, **11:** 487-499.

15. Naidoo B, Thorogood M, McPherson K, Gunning-Schepers LJ: **Modelling the effects of increased physical activity on coronary heart disease in England and Wales.** *J Epidemiol Community Health* 1997, **51:** 144-150.

16. Bronnum-Hansen H, Juel K: **Estimating mortality due to cigarette smoking: two methods, same result.** *Epidemiology* 2000, **11:** 422-426.

17. Mooy JM, Gunning-Schepers LJ: **Computer-assisted health impact assessment for intersectoral health policy.** *Health Policy* 2001, **57:** 169-177.

18. Bronnum-Hansen H: **Predicting the effect of prevention of ischaemic heart disease.** *Scandinavian Journal of Public Health* 2002, **30:** 5-11.

19. Grover SA, Abrahamowicz M, Joseph L, Brewer C, Coupal L, Suissa S: **The benefits of treating hyperlipidemia to prevent coronary heart disease. Estimating changes in life expectancy and morbidity.** *JAMA* 1992, **267:** 816-822.

20. Hamilton VH, Racicot FE, Zowall H, Coupal L, Grover SA: **The cost-effectiveness of HMG-CoA reductase inhibitors to prevent coronary heart disease. Estimating the benefits of increasing HDL-C.** *JAMA* 1995, **273:** 1032-1038.

21. Grover SA, Paquet S, Levinton C, Coupal L, Zowall H: **Estimating the benefits of modifying risk factors of cardiovascular disease: a comparison of primary vs secondary prevention.** *Archives of Internal Medicine* 1998, **158:** 655-662.

22. Perreault S, Hamilton VH, Lavoie F, Grover S: **Treating hyperlipidemia for the primary prevention of coronary disease. Are higher dosages of lovastatin cost-effective?[comment].** *Archives of Internal Medicine* 1998, **158:** 375-381.

23. Grover SA, Coupal L, Paquet S, Zowall H: **Cost-effectiveness of 3-hydroxy-3-methylglutaryl-coenzyme A reductase inhibitors in the secondary prevention of cardiovascular disease: forecasting the incremental benefits of preventing coronary and cerebrovascular events.** *Archives of Internal Medicine* 1999, **159:** 593-600.

24. Perreault S, Dorais M, Coupal L, Paradis G, Joffres MR, Grover SA: **Impact of treating hyperlipidemia or hypertension to reduce the risk of death from coronary artery disease.[erratum appears in CMAJ 1999 Jul 13;161(1):21].** *CMAJ Canadian Medical Association Journal* 1999, **160:** 1449-1455.

25. Lowensteyn I, Coupal L, Zowall H, Grover SA: **The cost-effectiveness of exercise training for the primary and secondary prevention of cardiovascular disease.** *Journal of Cardiopulmonary Rehabilitation* 2000, **20:** 147-155.

26. Grover SA, Coupal L, Zowall H, Alexander CM, Weiss TW, Gomes DR: **How cost-effective is the treatment of dyslipidemia in patients with diabetes but without cardiovascular disease?** *Diabetes Care* 2001, **24:** 45-50.

27. Grover SA, Ho V, Lavoie F, Coupal L, Zowall H, Pilote L: **The importance of indirect costs in primary cardiovascular disease prevention: can we save lives and money with statins?** *Archives of Internal Medicine* 2003, **163:** 333-339.

28. Babad H, Sanderson C, Naidoo B, White I, Wang D: **The development of a simulation model of primary prevention strategies for coronary heart disease.** *Health Care Management Science* 2002, **5:** 269-274.

29. Cooper K, Davies R, Roderick P, Chase D, Raftery J: **The development of a simulation model of the treatment of coronary heart disease.** *Health Care Manag Sci* 2002, **5:** 259-267.

30. Capewell S, Morrison CE, McMurray JJ: **Contribution of modern cardiovascular treatment and risk factor changes to the decline in coronary heart disease mortality in Scotland between 1975 and 1994.** *Heart* 1999, **81:** 380-386.

31. Capewell S, Pell JP, Morrison C, McMurray J: **Increasing the impact of cardiological treatments. How best to reduce deaths [see comments].** *Eur Heart J* 1999, **20:** 1386-1392.

32. Capewell S, Beaglehole R, Seddon M, McMurray J: **Explanation for the decline in coronary heart disease mortality rates in Auckland, New Zealand, between 1982 and 1993.** *Circulation* 2000, **102:** 1511-1516.

33. Critchley JA, Capewell S: **Substantial potential for reductions in coronary heart disease mortality in the UK through changes in risk factor levels.** *J Epidemiol Community Health* 2003, **57:** 243-247.

34. Critchley JA, Capewell S, Unal B: **Life-years gained from coronary heart disease mortality reduction in Scotland: prevention or treatment?** *J Clin Epidemiol* 2003, **56:** 583-590.

35. Murray CJ, Lopez AD: **Global mortality, disability, and the contribution of risk factors: Global Burden of Disease Study.** *Lancet* 1997, **349:** 1436-1442.

36. Murray CJ, Lopez AD: **Alternative projections of mortality and disability by cause 1990-2020: Global Burden of Disease Study.** *Lancet* 1997, **349:** 1498-1504.

37. Ezzati M, Lopez AD, Rodgers A, Vander HS, Murray CJ: **Selected major risk factors and global and regional burden of disease.** *Lancet* 2002, **360:** 1347-1360.

38. Murray CJ, Lauer JA, Hutubessy RC, Niessen L, Tomijima N, Rodgers A *et al*.: **Effectiveness and costs of interventions to lower systolic blood pressure and cholesterol: a global and regional analysis on reduction of cardiovascular-disease risk.** *Lancet* 2003, **361:** 717-725.

39. Kottke TE, Puska P, Salonen JT, Tuomilehto J, Nissinen A: **Projected effects of high-risk versus population-based prevention strategies in coronary heart disease.** *Am J Epidemiol* 1985, **121:** 697-704.

40. Nissinen A, Tuomilehto J, Kottke TE, Puska P: **Cost-effectiveness of the North Karelia Hypertension Program. 1972-1977.** *Med Care* 1986, **24:** 767-780.

41. Browner WS: **Estimating the impact of risk factor modification programs.** *American Journal of Epidemiology* 1986, **123:** 143-153.

42. Hjort PF, Waaler HT: **What is the economic impact of secondary prevention to society?** *European Heart Journal* 1986, **7 Suppl B:** 67-73.

43. Kottke TE, Gatewood LC, Wu SC, Park HA: **Preventing heart disease: is treating the high risk sufficient?** *J Clin Epidemiol* 1988, **41:** 1083-1093.

44. Park H: **A multistate model for coronary heart disease--an application to different prevention strategies.** *In'gu Pogon Nonjip* 1989, **9:** 159-174.

45. Martens LL, Rutten FF, Erkelens DW, Ascoop CA: **Clinical benefits and cost-effectiveness of lowering serum cholesterol levels: the case of simvastatin and cholestyramine in The Netherlands.** *American Journal of Cardiology* 1990, **65:** 27F-32F.

46. Johannesson M, Hedbrant J, Jonsson B: **A computer simulation model for cost-effectiveness analysis of cardiovascular disease prevention.** *Medical Informatics* 1991, **16:** 355-362.

47. Doliszny KM, Luepker RV, Burke GL, Pryor DB, Blackburn H: **Estimated contribution of coronary artery bypass graft surgery to the decline in coronary heart disease mortality: the Minnesota Heart Survey.** *Journal of the American College of Cardiology* 1994, **24:** 95-103.

48. Silagy C, Mant D, Carpenter L, Muir J, Neil A: **Modelling different strategies to prevent coronary heart disease in primary care.** *J Clin Epidemiol* 1994, **47:** 993-1001.

49. Bonneux L, Barendregt JJ, Meeter K, Bonsel GJ, Van der Maas PJ: **Estimating clinical morbidity due to ischemic heart disease and congestive heart failure: the future rise of heart failure.** *Am J Public Health* 1994, **84:** 20-28.

50. Bensley DC, Watson PS, Morrison GW: **Pathways of coronary care--a computer-simulation model of the potential for health gain.** *IMA Journal of Mathematics Applied in Medicine & Biology* 1995, **12:** 315-328.

51. Pharoah PD, Hollingworth W: **Cost effectiveness of lowering cholesterol concentration with statins in patients with and without pre-existing coronary heart disease: life table method applied to health authority population.[comment].** *BMJ* 1996, **312:** 1443-1448.

52. Oster G, Thompson D: **Estimated effects of reducing dietary saturated fat intake on the incidence and costs of coronary heart disease in the United States.** *Journal of the American Dietetic Association* 1996, **96:** 127-131.

53. Bots ML, Grobbee DE: **Decline of coronary heart disease mortality in The Netherlands from 1978 to 1985: contribution of medical care and changes over time in presence of major cardiovascular risk factors.** *J Cardiovasc Risk* 1996, **3:** 271-276.

54. Lightwood JM, Glantz SA: **Short-term economic and health benefits of smoking cessation: myocardial infarction and stroke.[comment].** *Circulation* 1997, **96:** 1089-1096.

55. Kellett J: **Likely gains in life expectancy of patients with coronary artery disease treated with HMG-CoA reductase inhibitors, as predicted by a decision analysis model.** *European Journal of Surgery* 1997, **163:** 539-546.

56. Riviere M, Wang S, Leclerc C, Fitzsimon C, Tretiak R: **Cost-effectiveness of simvastatin in the secondary prevention of coronary artery disease in Canada.** *CMAJ* 1997, **156:** 991-997.

57. Bonneux L, Barendregt JJ, Nusselder WJ, der Maas PJ: **Preventing fatal diseases increases healthcare costs: cause elimination life table approach.** *BMJ* 1998, **316:** 26-29.

58. Galgali G, Beaglehole R, Scragg R, Tobias M: **Potential for prevention of premature death and disease in New Zealand.** *New Zealand Medical Journal* 1998, **111:** 7-10.

59. Augustovski FA, Cantor SB, Thach CT, Spann SJ: **Aspirin for primary prevention of cardiovascular events.** *Journal of General Internal Medicine* 1998, **13:** 824-835.

60. Bonneux L, Barendregt JJ, Van der Maas PJ: **The new old epidemic of coronary heart disease.** *Am J Public Health* 1999, **89:** 379-382.

61. Mui S-L: **Projecting coronary heart disease incidence and cost in Australia: Results from the Incidence module of the Cardiovascular Disease Policy Model.** *Australian and New Zealand Journal of Public Health, Vol 23(1) (pp 11-19), 1999*.

62. Thompson D, Edelsberg J, Colditz GA, Bird AP, Oster G: **Lifetime health and economic consequences of obesity.** *Archives of Internal Medicine* 1999, **159:** 2177-2183.

63. Lindholm L, Hallgren CG, Boman K, Markgren K, Weinehall L, Ogren JE: **Cost-effectiveness analysis with defined budget: how to distribute resources for the prevention of cardiovascular disease?** *Health Policy* 1999, **48:** 155-170.

64. Naidoo B, Stevens W, McPherson K: **Modelling the short term consequences of smoking cessation in England on the hospitalisation rates for acute myocardial infarction and stroke.** *Tob Control* 2000, **9:** 397-400.

65. Kuulasmaa K, Tunstall PH, Dobson AJ, France M, Sans S, Tolonen H *et al*.: **Estimation of contribution of changes in classic risk factors to trends in coronary-event rates across the WHO MONICA Project.** *Lancet* 2000, **355:** 675-687.

66. Fichtenberg C, Glantz S: **Association of the California Tobacco Control Program with Declines in Cigarette Consumption and Mortality from Heart Disease.** *N Engl J Med* 2001, **343:** 1772-1777.

67. McNeil JJ, Peeters A, Liew D, Lim S, Vos T: **A model for predicting the future incidence of coronary heart disease within percentiles of coronary heart disease risk.** *Journal of Cardiovascular Risk* 2001, **8:** 31-37.

68. Baker S, Priest P, Jackson R: **Using thresholds based on risk of cardiovascular disease to target treatment for hypertension: modelling events averted and number treated.** *BMJ* 2000, **320:** 680-685.

69. Selmer RM, Kristiansen IS, Haglerod A, Graff-Iversen S, Larsen HK, Meyer HE *et al*.: **Cost and health consequences of reducing the population intake of salt.** *Journal of Epidemiology & Community Health* 2000, **54:** 697-702.

70. Malik IS, Bhatia VK, Kooner JS: **Cost effectiveness of ramipril treatment for cardiovascular risk reduction.** *Heart (British Cardiac Society)* 2001, **85:** 539-543.

71. Peeters A, Mamun AA, Willekens F, Bonneux L: **A cardiovascular life history. A life course analysis of the original Framingham Heart Study cohort.** *Eur Heart J* 2002, **23:** 458-466.

72. Marshall T, Rouse A: **Resource implications and health benefits of primary prevention strategies for cardiovascular disease in people aged 30 to 74: mathematical modelling study.[comment][erratum appears in BMJ 2002 Oct 5;325(7367):756].** *BMJ* 2002, **325:** 197.

73. Assmann G, Schulte H: **Modelling the Helsinki Heart Study by means of risk equations obtained from the PROCAM Study and the Framingham Heart Study.** *Drugs* 1990, **40 Suppl 1:** 13-18.

74. Avins AL, Browner WS: **Improving the prediction of coronary heart disease to aid in the management of high cholesterol levels: what a difference a decade makes.** *JAMA* 1998, **279:** 445-449.

75. Beard CM, Kottke TE, Annegers JF, Ballard DJ: **The Rochester Coronary Heart Disease Project: effect of cigarette smoking, hypertension, diabetes, and steroidal estrogen use on coronary heart disease among 40- to 59-year-old women, 1960 through 1982.** *Mayo Clin Proc* 1989, **64:** 1471-1480.

76. Bronnum-Hansen H: **How good is the Prevent model for estimating the health benefits of prevention?** *J Epidemiol Community Health* 1999, **53:** 300-305.

77. Cleland JG, Walker A: **Therapeutic options and cost considerations in the treatment of ischemic heart disease.** *Cardiovascular Drugs & Therapy* 1998, **12 Suppl 3:** 225-232.

78. Cowen-ME, Bannister-M, Shellenberger-R, Tilden-R: **A guide for planning community-oriented health care: the health sector resource allocation model.** *Med-Care* 1996, **34:** 264-279.

79. Glick H, Heyse JF, Thompson D, Epstein RS, Smith ME, Oster G: **A model for evaluating the cost-effectiveness of cholesterol-lowering treatment.** *Int J Technol Assess Health Care* 1992, **8:** 719-734.

80. Grover SA, Coupal L: **Risk-benefit assessment of drug treatment to prevent coronary heart disease. Estimating the benefits of risk factor modification. [Review] [23 refs].** *Drug Safety* 1994, **10:** 301-309.

81. Gunning-Schepers LJ: **Models: instruments for evidence based policy.** *J Epidemiol Community Health* 1999, **53:** 263.

82. Gunning-Schepers LJ, Hagen JH: **Avoidable burden of illness: how much can prevention contribute to health?** *Soc Sci Med* 1987, **24:** 945-951.

83. Hatziandreu EI, Koplan JP, Weinstein MC, Caspersen CJ, Warner KE: **A cost-effectiveness analysis of exercise as a health promotion activity.** *American Journal of Public Health* 1988, **78:** 1417-1421.

84. Hinzpeter B, Klever-Deichert G, Wendland G, Lauterbach KW: **[Coronary disease and social security. A simulation model on cost analysis].** *Herz* 2000, **25:** 515-525.

85. Kaplan GA, Cohn BA, Cohen RD, Guralnik J: **The decline in ischemic heart disease mortality: prospective evidence from the Alameda County Study.** *American Journal of Epidemiology* 1988, **127:** 1131-1142.

86. Kawachi I, Purdie G: **Should treatment of mild to moderate hypertension be targeted? Results from a Markov cohort model incorporating multiple risk factors.** *Journal of Human Hypertension* 1990, **4:** 651-658.

87. Kaplan RM, Ake CF, Emery SL, Navarro AM: **Simulated effect of tobacco tax variation on population health in California.** *Am J Public Health* 2001, **91:** 239-244.

88. Liew D, McNeil JJ, Peeters A, Lim SS, Vos T: **Epidemiological modelling (including economic modelling) and its role in preventive drug therapy.** *Medical Journal of Australia* 2002, **177:** 364-367.

89. Murray DM, Hannan PJ, Jacobs DR, McGovern PJ, Schmid L, Baker WL *et al*.: **Assessing intervention effects in the Minnesota Heart Health Program.** *American Journal of Epidemiology* 1994, **139:** 91-103.

90. Murray CJ, Lopez AD, Jamison DT: **The global burden of disease in 1990: summary results, sensitivity analysis and future directions.** *Bull World Health Organ* 1994, **72:** 495-509.

91. Murray CJ, Lopez AD: **Mortality by cause for eight regions of the world: Global Burden of Disease Study.** *Lancet* 1997, **349:** 1269-1276.

92. Nissinen A, Tuomilehto J, Enlund H, Kottke TE: **Costs and benefits of community programmes for the control of hypertension.** *J Hum Hypertens* 1992, **6:** 473-479.

93. Peeters A, Barendregt JJ, Willekens F, Mackenbach JP, Al Mamun A, Bonneux L: **Obesity in adulthood and its consequences for life expectancy: a life-table analysis.** *Ann Intern Med* 2003, **138:** 24-32.

94. Petersen CC: **Simulation of alternative designs of a cardiovascular risk reduction program for the U.S. Air Force.** *Journal of Medical Systems* 1982, **6:** 149-164.

95. Salonen JT, Kottke TE, Jacobs DR, Jr., Hannan PJ: **Analysis of community-based cardiovascular disease prevention studies--evaluation issues in the North Karelia Project and the Minnesota Heart Health Program.** *International Journal of Epidemiology* 1986, **15:** 176-182.

96. Wolfson MC: **POHEM- a framework for understanding and modelling the health of human populations.** *World Health Stat Q* 1994, **47:** 157-176.
